# Political diversity in U.S. police agencies

**DOI:** 10.1111/ajps.12945

**Published:** 2025-02-14

**Authors:** Bocar Ba, Haosen Ge, Jacob Kaplan, Dean Knox, Mayya Komisarchik, Gregory Lanzalotto, Rei Mariman, Jonathan Mummolo, Roman Rivera, Michelle Torres

**Affiliations:** 1 Department of Economics, Duke University, Social Sciences Building, Durham, North Carolina, USA; 2 Wharton AI & Analytics Initiative, Analytics at Wharton, University of Pennsylvania, Philadelphia, Pennsylvania, USA; 3 School of Public and International Affairs, Princeton University, Princeton, New Jersey, USA; 4 Operations, Information and Decisions Department, University of Pennsylvania, Philadelphia, Pennsylvania, USA; 5 Department of Political Science, University of Rochester, Rochester, New York, USA; 6 Operations, Information and Decisions Department, University of Pennsylvania, Philadelphia, Pennsylvania, USA; 7 Data Computing and Research Support, Analytics at Wharton, University of Pennsylvania, Philadelphia, Pennsylvania, USA; 8 Department of Politics, School of Public and International Affairs, Princeton University, Princeton, New Jersey, USA; 9 Institute for Research on Labor and Employment, University of California, Berkeley, California, USA; 10 Department of Political Science, University of California, Los Angeles, California, USA

## Abstract

Partisans are divided on policing policy, which may affect officer behavior. We merge rosters from 99 of the 100 largest local U.S. agencies—over one third of local law enforcement agents nationwide—with voter files to study police partisanship. Police skew more Republican than their jurisdictions, with notable exceptions. Using fine-grained data in Chicago and Houston, we compare behavior of Democratic and Republican officers facing common circumstances. We find minimal partisan differences after correcting for multiple comparisons. But consistent with prior work, we find Black and Hispanic officers make fewer stops and arrests in Chicago, and Black officers use force less often in both cities. Comparing same-race partisans, we find White Democrats make more violent crime arrests than White Republicans in Chicago. Our results suggest that despite Republicans’ preference for more punitive law enforcement policy and their overrepresentation in policing, partisan divisions often do not translate into detectable differences in on-the-ground enforcement.

Policing has become a locus of partisan strife in the United States (Eckhouse, 2019; Parker & Hurst, 2021; Grosjean, Masera, & Yousaf, 2023). Republicans are far more likely than Democrats to trust police, more likely to believe police treat different groups equally, less likely to think police killings are a problem, and less likely to think Black Lives Matter protests are motivated by a genuine desire to hold police accountable (Pew, 2016). In fact, as we show below, party identification is among the most important individual-level predictors of policing-related attitudes, surpassing the importance of race or political ideology (see Figure 1 and accompanying discussion).

While partisans in the general public may disagree strongly about how police should function in society, few are empowered to translate their political views into action. Police officers experience no such constraint. Every day, armed agents of the state are deployed in American communities with extraordinary discretion over whether, when, and how to enforce the law (Wilson, 1968; Goldstein, 1977). It is no exaggeration to note that police officers often have the ability to make policing policy unilaterally in real time (Lipsky, 1980). This power, combined with sharp partisan divisions over how police should do their jobs, raises several important questions that speak not only to the determinants of police behavior but to the health of democratic representation (Kingsley, 1944; Meier, 1975). What share of police identify with the Republican and Democratic parties? To what extent do these identities reflect those of the local civilians whom police serve? And how do officers with differing partisan affiliations behave when interacting with those civilians?

Progress on these questions has been hampered by an incomplete and heterogeneous landscape of administrative data. Assembling basic facts about law enforcement agents remains remarkably difficult in many jurisdictions. Agencies rarely share information proactively and sometimes defy the near-universal requirement to disclose government employee rosters under freedom-of-information laws. In light of these obstacles, researchers typically turn to one of two alternatives. The first is to closely study single jurisdictions (Ba et al., 2021; Hoekstra & Sloan, 2020; Donahue, 2023), leaving open questions of generalizability. Alternatively, researchers have conducted national surveys of police officers (Morin et al., 2017), but because these studies often sample small numbers of officers from numerous locations nationwide, they preclude examination of whether and how agencies represent their particular jurisdictions, especially in terms of political views and affiliations. In addition, survey-based methods are prone to severe selection bias, since many officers (and even entire police agencies) decline participation.^1^

In this paper, we analyze nearly a quarter million officers, covering 99 of the 100 largest local U.S. agencies and representing over one third of all local law enforcement agents nationwide, to study officers’ partisan affiliations. Our data draw upon numerous open records requests, data-sharing agreements, and publicly available personnel rosters, merged with voter files and U.S. Census data. In addition to party identification, our data contain measures of officers’ race/ethnicity, gender, age, income, voting history, and place of residence, allowing us to comprehensively characterize the degree to which police resemble their communities on a host of dimensions as well as how this correspondence varies across jurisdictions. In addition, micro-level data on officers’ day-to-day deployment and enforcement behaviors in two of the five largest local police forces in the United States—the Chicago Police Department (CPD) and the Houston Police Department (HPD)—allow us to carefully examine whether Democratic and Republican officers behave differently when facing common circumstances.

We use these data to address classic questions in the literature on “representative bureaucracy” (RB) (Kingsley, 1944; Dolan & Rosenbloom, 2003), which holds that bureaucrats sharing salient social identities with civilians will offer superior service under some conditions. We first conduct the most comprehensive analysis to date of “passive representation” (PR) in policing: an assessment of whether bureaucrats resemble the civilians they serve on various dimensions (Meier, 2019). We demonstrate that relative to civilians in their jurisdictions, police officers are not only more likely to affiliate with the Republican Party, they also have higher household income, vote more often, and are more likely to be White. However, the degree of unrepresentativeness is heterogeneous, with some agencies closely mirroring their populations and others substantially diverging.^2^

To probe the behavioral consequences of these patterns at a finer-grained level, we turn to our micro-level data in Chicago and Houston. Chicago represents a crucial case for the study of diversity in policing (McCrary, 2007): the agency has substantially diversified along racial, ethnic, and gender lines in recent decades; the city remains a focal point for concerns over abusive policing practices; and public opinion polls there show sharp divergences between racial and ethnic groups of civilians on attitudes towards police (Harris, 2021). While HPD has also been criticized for racial disparities in policing outcomes (deGrood, 2023; Vasquez, 2023), it differs in an important respect—its ranks are roughly balanced between Democrats and Republicans, unlike CPD’s heavily Democratic makeup. By analyzing the dynamics of police–civilian interactions across differing contexts, we can begin to move beyond the tendency in this literature to examine officer behavior in single jurisdictions, which is severely limiting in the U.S. federalist context.

Both our Chicago and Houston data include the precincts to which police officers are assigned, allowing us to evaluate a more specific form of PR: whether officers resemble civilians in the areas they patrol. We see striking gaps in political affiliation: every single district in Chicago and nearly every division in Houston is policed by officers who skew more Republican than local residents.

Having established these descriptive patterns, we then use data on CPD and HPD daily assignments and enforcement records to investigate how officers’ partisan identities map to behavior on the job. In other words, we probe for indications that police officers of various backgrounds practice “active representation” (AR), behaving in ways that accord with the preferences of civilians who are passively represented (Meier, 2019, p. 40). While this analysis is limited to two cities, we focus on them because it allows for the most credible test to date of behavioral differences between officers of differing political identities. As we explain in detail below, the incorporation of shift assignment data lets us address a key limitation in prior studies that link officer partisanship to behavior (e.g., Donahue, 2023) by allowing us to compare officers assigned to police comparable pools of civilians in comparable situations. This avoids the selection issues that prior work has shown can produce severe bias when analyzing enforcement data alone—e.g., selectively analyzing only the subset of situations where officers chose to make stops or issue citations (Knox, Lowe, & Mummolo, 2020; Ba et al., 2021).

Specifically, we estimate differences in the overall numbers of stops, arrests, and uses of force made by Democratic and Republican officers. We further examine the amount of enforcement directed toward various civilian racial groups and involving various types of arrests. Comparisons are made between Republican and Democratic officers in the aggregate as well as between Republican and Democratic officers of the same race. Each test compares officers deployed to comparable places, times, and tasks, ensuring officer behavior is always evaluated against behavior by peers facing common circumstances.

In brief, we find few detectable differences across partisan groups after correcting for multiple comparisons. However, consistent with prior work (Ba et al., 2021), we find Black and Hispanic officers make fewer stops and arrests in Chicago, and Black officers use force less often in both cities. Among White officers in Chicago, Democrats make more arrests for violent crime than Republicans. Within other racial groups, Democratic and Republican behavior is statistically indistinguishable after multiple-testing corrections.

Taken together, our results provide new insight into how officers’ social identities map to those of the civilians they serve, as well as officers’ behavior during interactions with civilians. While police certainly skew Republican and White overall, there exist agencies where both the partisan and racial compositions of the force closely mirror the population at large, such as the Birmingham, AL, Police Department. And though partisans disagree on how policing should be conducted (Pew, 2020), those divisions do not generally correspond to Democratic–Republican differences in officer behavior. Finally, where partisan differences can be found, Democrats are more active than Republicans in their enforcement—diverging from partisan preferences on policing policy in the general population.

## EMPIRICAL ASSESSMENTS OF DIVERSITY IN POLICING

A large interdisciplinary literature has investigated whether police officers demographically resemble the civilians they serve as well as whether various officer attributes and identities are systematically related to behavior on the job. The vast majority of this work focuses on race and gender. As previous reviews note, these studies have produced mixed results, especially with respect to police behavior (Sklansky, 2005). At least part of this apparent disagreement is due to the use of incomplete data sources and analytic approaches later shown to be vulnerable to selection bias.

Many earlier studies of diversity in law enforcement focused on cross-sectional comparisons, such as agency-level correlations showing whether diversity was associated with various aggregate outcomes. For example, Meier and Nicholson-Crotty (2006) finds that agencies with a higher percentage of female officers tend to see more sexual assault reports and arrests. Similarly, Wilkins and Williams (2008) finds that “the presence of black police officers [in an agency’s division] is related to an increase in racial profiling in the division.” The well-known concern with this class of studies is that police agencies—and divisions within single agencies—differ immensely in unobserved ways that correlate with both diversity and these outcomes, posing the strong risk of omitted variable bias.

A more recent set of studies has leveraged incident-level data to compare the post-stop enforcement actions of various officer groups, using data on civilians who were stopped. In an analysis of officers’ decisions to search stopped motorists, Baumgartner et al. (2021) finds that across officers of all racial groups, stops of Black male civilians lead to searches more often than any other civilian demographic. The study also found searches made by White male officers were less likely to lead to an arrest. In a related study, Shoub, Stauffer, and Song (2021) examines traffic stops in two agencies and find “female officers are less likely to search drivers than men,” but “when female officers do conduct a search, they are more likely to find contraband and they confiscate the same net amount of contraband as male officers” (p. 1).

Analyzing close election outcomes for sheriff, Thompson (2020) shows that Democrats and Republicans comply with federal requests relating to immigration enforcement at comparable rates. Most relevant to the current study is Donahue (2023), which merges data on Florida Highway Patrol traffic stops with voter records and finds “White Republican officers exhibit a larger racial disparity than White Democratic officers in their propensity to search motorists whom they have stopped” (p. 1).

These studies make important contributions, but they also each exhibit a common limitation: data are limited to police–civilian encounters in which officers choose to initiate a stop. As Donahue (2023) itself acknowledges, this makes the conclusions vulnerable to selection bias (pp. 664–665).^3^ Previous research has established that neglecting selection issues in police administrative records can distort inferences in complex ways (Knox, Lowe, & Mummolo, 2020). Intuitively, this is because if some groups of officers discriminate by stopping e.g. minorities in less severe circumstances, and those circumstances are not fully documented in police records (and therefore cannot be adjusted for), then minority stops will not be comparable to White stops despite being seemingly identical on officer-reported characteristics.

For studies that seek to estimate the frequency with which officers take actions against civilians (e.g., how often Black officers make arrests), it is thus crucial to account in some way for the denominator of all opportunities that were available for that action to be taken—not only the stops that were *actually* made but also encounters in which civilians were allowed to pass freely (Knox & Mummolo, 2020). This detailed encounter-level data is rarely available for non-stops. To address this issue, a viable workaround is to use data on the places and times where officers are deployed, because researchers can infer that officers assigned to work in common circumstances will be faced with the same pool of encounters where action could be taken, even if those encounters cannot be directly observed themselves (Ba et al., 2021). But without this deployment data on the precise places and times where officers work—or research designs that can render time and place ignorable—a serious challenge arises. Constructing the correct denominator for enforcement rates becomes fraught, and behavioral differences between two groups of officers become difficult to disentangle from contextual differences in the types of assignments faced by the two groups.

Some recent studies have made progress in overcoming these challenges with deployment data. Using micro-level data in Chicago on officer shift assignments and behavior, and leveraging exogenous variation in rotating day-off schedules, Ba et al. (2021) finds deploying officers of color (relative to White officers) or female officers (relative to male officers) to otherwise similar circumstances leads to substantial reductions in stops, arrests, and uses of force. Using data on dispatches to 911 calls within specific places and times, Hoekstra and Sloan (2020) finds that “while white and black officers use gun force at similar rates in white and racially mixed neighborhoods, white officers are five times as likely to use gun force in predominantly black neighborhoods” (p. 1). And leveraging the quasi-random assignment of officers to the scene of traffic accidents, West (2018) finds “officers issue significantly more traffic citations to drivers whose race differs from their own” (p. 1). In this paper, we extend the approach of Ba et al. (2021) to the study of officer partisanship in the research design section.

## REPRESENTATIVE BUREAUCRACY AND PARTISAN IDENTITY

In this section, we draw on established literature on RB and partisan polarization to theorize about the ways in which partisan identification might influence police behavior. Calls to diversify police forces represent perhaps the oldest proposed policing reform, and one argument for diversification springs from the literature on RB. RB theories (Kingsley, 1944; Dolan & Rosenbloom, 2003) are premised on several assertions: bureaucratic oversight is often incapable of ensuring that bureaucrats will exercise discretion in desirable ways (Huber & Shipan, 2002); staffing agencies with workers who share values with the population at large will promote desirable outputs (Bendor & Meirowitz, 2004); and observable worker traits, often standard demographic indicators, are useful proxies for shared values (Meier, 1975).

A key precondition for RB is PR, which describes the degree to which bureaucrats mirror their clients on a given attribute or identity. In this paper, we shed light on the extent of PR by assessing correspondence between civilian and officer traits across 99 of the largest 100 police agencies in the United States. However, the mere existence of PR does not guarantee AR: “cases where the bureaucracy produces benefits for the clients passively represented” (Meier, 2019, p. 40). Over the years, RB scholars have posited various conditions under which bureaucrats are more likely to engage in AR. In this work, we assess AR in policing through a behavioral analysis that examines how partisanship and race simultaneously map to police behavior.

Prior work has theorized that AR is more likely to occur when the salience of a relevant identity increases (Meier, Pennington, & Eller, 2005). The intensifying political polarization surrounding policing policy raises the possibility that partisan identity—which has grown more prominent in society generally (Iyengar & Westwood, 2015)—may be playing an increased role in how officers perform their day-to-day duties. Prior work on affective polarization offers several reasons why partisan affiliations might affect police behavior. For one, “partisanship has bled into the nonpolitical sphere, driving ordinary citizens to reward copartisans and penalize opposing partisans” (Iyengar et al., 2019, p. 133) in arenas as varied as hiring (Gift & Gift, 2015), dating (Huber & Malhotra, 2017), and online labor markets (McConnell et al., 2018). Recent evidence from public administration also shows that bureaucrats who run elections respond differently to voters’ information requests when voters disclose their partisanship (Porter & Rogowski, 2018).

One potential obstacle to partisan AR in policing is that, unlike other demographic characteristics, a civilian’s partisanship is not readily observable by most officers, perhaps making it more difficult to actively provide preferential treatment. However, we theorize that there are at least two ways that partisan AR can still occur. First, recent experimental work has shown that racial stimuli can activate partisan animus and vice versa (Westwood & Peterson, 2022). And because partisan divisions on policing policy are so strongly tied to matters of race, officers may actively represent copartisans indirectly through their treatment of various civilian racial groups. Consistent with this logic, Grosjean, Masera, and Yousaf (2023) shows that police are more likely to stop Black drivers in the wake of Trump rallies—events where Trump has explicitly downplayed police brutality (Eversley, 2017). Second, in the realm of policing, civilians can accrue “benefits” from officers who share their social identity without directly interacting with those officers. For example, officers can suppress a certain type of crime that is of principal concern to in-group members. By behaving in ways consistent with copartisans’ views on how policing should be done, officers can provide AR for their partisan group without identifying or knowingly interacting with individual copartisans.

**FIGURE 1:**
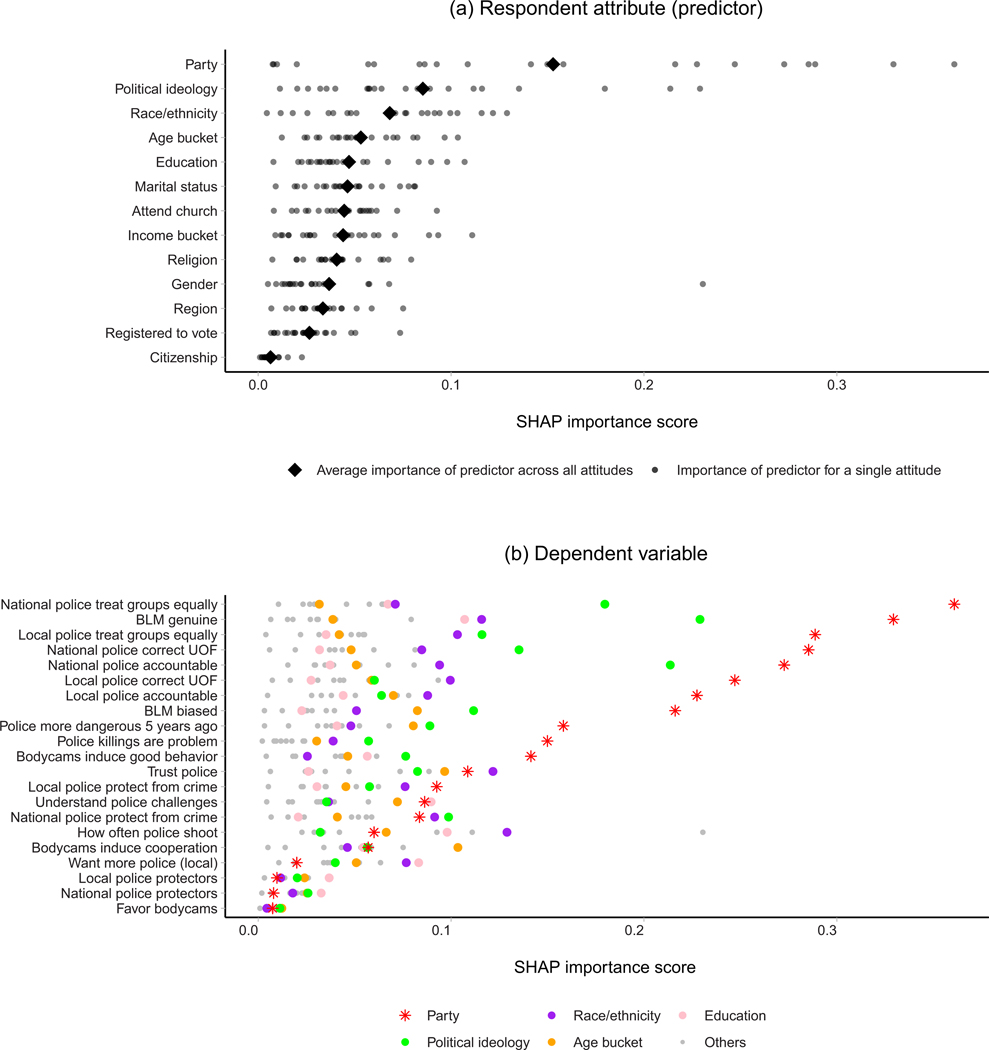
Partisanship as a predictor of policing attitudes. *Note*: The upper panel depicts the Shapley additive explanation importance score (SHAP importance, horizontal axis) of various respondent attributes (vertical axis) in predicting survey responses about policing in Pew (2016). Each small gray circle represents a policing attitude, with a vertical position indicating the attribute’s contribution to overall estimated responses (Amoukou, Brunel, and Tangi, 2022) in a gradient-boosted decision tree model (Chen & Guestrin, 2016). Large black diamonds represent the overall importance of the respondent attribute, averaging over all attitudes. Partisanship has the highest overall importance, roughly double that of ideology and race/ethnicity. The lower panel shows disaggregated importance scores (horizontal axis) for each policing attitude (vertical axis) with points for each respondent attribute. Partisanship is indicated with a red asterisk and other top-five predictors are indicated by colored dots; for clarity, less important attributes are shown only with gray dots. Partisanship is the most important predictor for a majority of policing attitudes.

The most obvious reason that officers of differing partisan identities might perform their jobs differently stems from public opinion data. National polls show clear evidence of partisan divides on a range of questions pertaining to how police should do their jobs. In Figure 1, we present the importance of partisan affiliation and other demographics in predicting policing attitudes in a national survey (Pew, 2016). The importance of each variable is estimated through its Shapley value, a standard machine learning technique that assesses how much predictions change when the variable is omitted.^4^ As the figure shows, partisan affiliation is among the most important predictors of policing attitudes, often eclipsing the predictive power of standard demographic variables including race/ethnicity and political ideology. If partisans in the general public mirror the preferences of partisans on police forces, it is plausible that these groups of officers behave in very different ways on the job. While we cannot directly measure officers’ preferences, our analysis below examines the distribution and consequences of police partisanship to assess whether patterns are consistent with AR.

In what follows, we discuss our empirical strategies for assessing the distribution and consequences of police officers’ partisan affiliations.

## DATA AND MEASUREMENT

We sought rosters of all sworn police officers in the largest 100 police agencies^5^ in the United States. We define “largest” based on the number of officers whose primary duty is patrol, as these officers are the ones most likely to have contact with members of the public (Harrell & Davis, 2020). We assembled data on 50 agencies by scouring public sources such as open-data portals managed by local governments, news agencies, and nonprofits, as well as data previously released through public records requests on muckrock.com. We obtained the remainder from a combination of open-records requests and data-sharing agreements. Roughly three quarters of rosters come from 2019 to 2021; about one fifth originate from 2015 to 2018; and the remainder do not specify a year.

Ultimately, we received data covering roughly 220,000 officers from 99 police agencies.^6^ In 91 agencies, we also obtained employee titles, which we use to distinguish sworn police officers and unsworn civilian roles (such as lab technicians and analysts). This information allows us to subset to sworn officers for much of our analysis.

**FIGURE 2:**
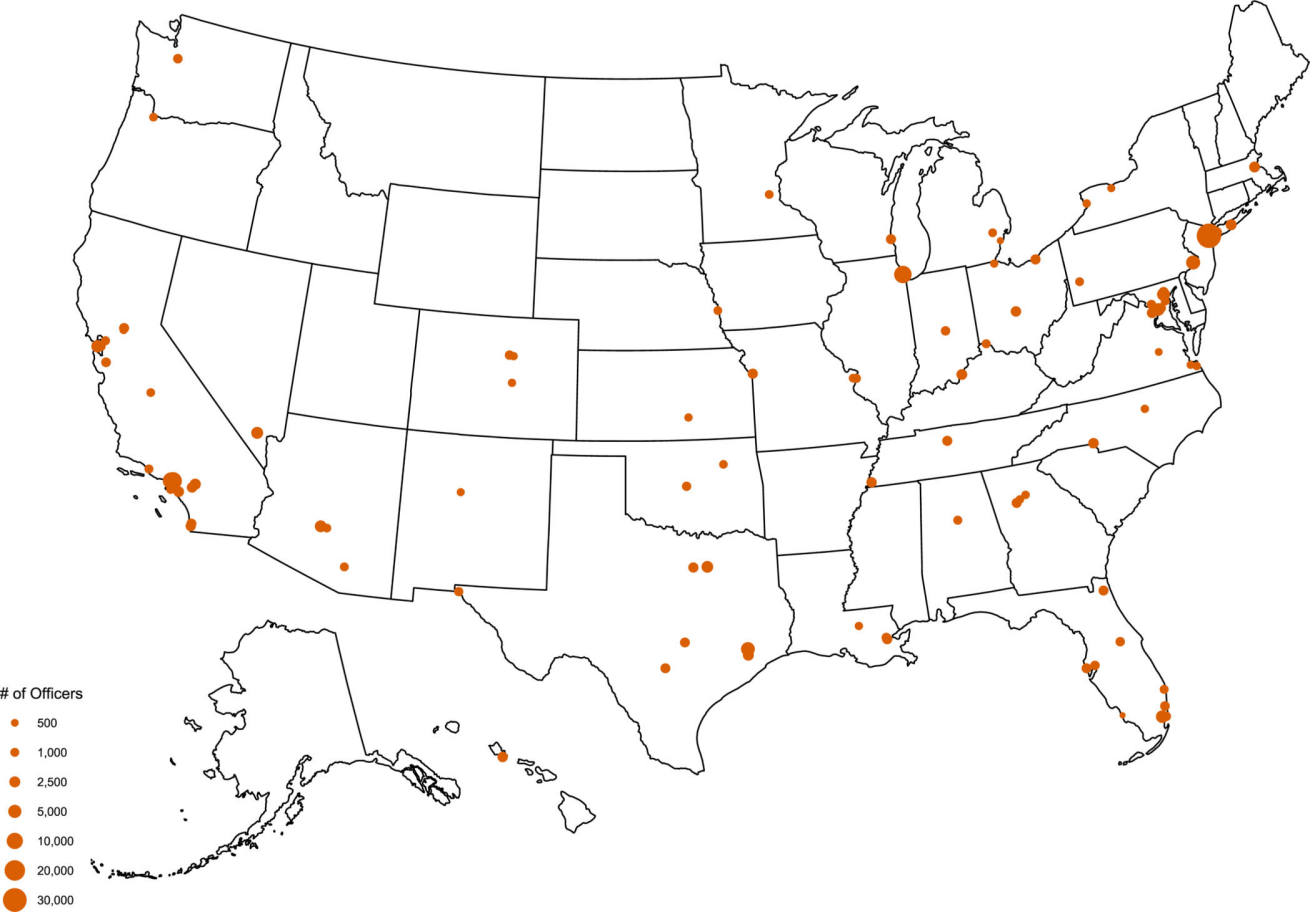
Agency locations. *Note*: Included agencies cover roughly 220,000 officers across 32 states and Washington, D.C., representing 34% of the nation’s roughly 642,000 sworn local police officers and sheriffs’ deputies (Hyland & Davis, 2019). Together, jurisdictions covered in our data serve about 23% of the U.S. population. Each dot is scaled by the number of sworn officers.

Figure 2 shows the location of each agency included in this study. Our data cover agencies in 32 states and the District of Columbia. In all, the roughly 220,000 officers in our agency rosters represent over one third of the roughly 642,000 local police officers and sheriffs’ deputies nationwide (Hyland & Davis, 2019), making this the largest examination of descriptive representation in policing to date.^7^

### Measuring officer attributes

Employee rosters contain full officer names, with the exception of a limited number of undercover agents in certain jurisdictions who are excluded from analysis. For our analysis comparing agencies to civilians in their jurisdictions (see the following section), we measure officer attributes with a combination of sources. We use voter file estimates to quantify party identification, turnout, age, and household income for individual officers, which we then aggregate to the agency level. For officer race and gender, we rely on agency responses to federal surveys, avoiding the estimated voter file proxies. In our behavioral analysis of Chicago and Houston, we use voter file measures of party identification but rely on individual-level racial data obtained through open-records requests.

We merge officer rosters with a commercial voter file from L2 (l2-data.com) via a two-step process. We restricted candidate matches to only individuals residing in or adjacent to the counties containing their agency, including adjacent out-of-state counties. We then attempted to find a match for each officer in our roster based on the officer’s first name, their middle initial (if available), and their last name. Rather than using exact matching, we employ a probabilistic technique (Enamorado, Fifield, & Imai (2017); Enamorado, Fifield, & Imai (2019)) via the *fastlink* R package.^8^ See Online Appendix Sections B (Appendix pp. 1–2) and I (Appendix pp. 15–24) for details on our matching procedure and extensive validation tests, respectively.

Data in the L2 voter file includes party identification, age, household income, and voter turnout history for both officers and civilians in their jurisdictions. We use these covariates, along with 2015–2019 five-year American Community Survey data, to evaluate PR.^9^ We divide officers and civilians into three partisan categories based on L2’s labels: Democrat, Republican, and an “other/unknown party” category that represents all other party affiliations in L2 along with all individuals not appearing in the L2 data. These categories rely on proprietary L2 algorithms to characterize the party affiliation of officers and civilians, which introduces potential bias due to errors in machine learning based proxies (Knox, Lucas, & Cho, 2022). While errors in these imputations may bias estimated levels of party affiliation, at least some of this bias would likely wash out when computing *differences* between officers and civilians because the same imputation method is applied to both groups. In addition, several studies have sought to validate L2’s imputed partisanship measures and found they correlate strongly to both official election returns (Fraga, Holbein, & Skovron, 2018) and self-reports in surveys.^10^ Studies of another potential source of error in voter files, so-called “insincere” party registration by partisans seeking to sabotage their opponents, have found virtually no evidence of the phenomenon (Stephenson, 2011).

Nevertheless, to address these concerns, we take extensive steps in Online Appendix I (Appendix pp. 15–24) to deal with potential measurement error in party identification: we compute bounds using extreme assumptions about covariates of unobserved individuals; we re-compute core results using an alternate measure of party identification; and we report results using only states in which voters can identify their preferred political party when registering to vote, where party identification data may be most accurate. Our core conclusions—e.g., about the overrepresentation of Republican and White identities in policing—remain supported across nearly all of these robustness checks.^11^

To measure race, ethnicity, and gender, we primarily rely on 2021 Law Enforcement Officers Killed and Assaulted (Kaplan, 2023, LEOKA, which reports gender breakdowns for officers in each reporting agency) and 2020 Law Enforcement Management and Administrative Statistics data (LEMAS, 2020, agency surveys reporting racial composition). These datasets contain demographic information on 100% and 86% of the agencies in our study, respectively. For missing agencies, we rely on L2’s estimated race and ethnicity. We similarly rely on L2 for measures of officers’ household income and age. See Online Appendix C (Appendix p. 2) and Online Appendix H (Appendix p. 15) for additional details.^12^

## OFFICERS’ POLITICAL AFFILIATIONS IN LOCAL CONTEXT

We now compare the partisan affiliations of officers to those of civilians within their jurisdictions. We also characterize descriptive representation of civilians on additional dimensions including race, ethnicity, gender, household income, age, and political participation as measured by general election turnout. Civilian attributes are obtained by aggregating over all Census tracts where the agency has jurisdiction.^13^

Table 1 first displays aggregate results. The leftmost values represent average officer attributes, aggregating across our 99 jurisdictions.^14^ Because each officer is given equal weight, larger agencies account for a larger share of these aggregate statistics; results disaggregated by agency are given in Online Appendix Table F.1 (Appendix pp. 7–9). The next column corresponds to the expected attribute value if, hypothetically, police agencies were perfectly representative—for example, the expected proportion of Republican officers across the 99 agencies, if each current officer was instead replaced with a random draw from their respective jurisdiction while holding agency sizes fixed.^15^ Subsequent columns display officer–civilian differences and 95% confidence intervals.^16^

Results show police officers diverge from their jurisdictions on every attribute we measure. We find officers are far more likely to be Republican than civilians in their jurisdictions: we estimate 32% of officers are Republican (vs. 14% of civilians in the voting-age population). Officers are also less likely to identify with the Democratic party (31%, vs. 44%), and officers are much more politically active (69% voted in the 2020 general election, vs. 55%).

In terms of race, 51% of officers in our data are White. If officers were representative of civilians in their jurisdictions, that share would fall to 38%; correspondingly, the Black and Hispanic proportion would rise by about 5 and 4 percentage points (p.p.), respectively. By far the largest representation gap is in gender: 83% of officers in our data are male, likely due in part to the difficulty of recruiting female candidates into law enforcement (Kringen, 2014). This gap is especially noteworthy given recent research showing that, when faced with common circumstances, female officers are less likely to use force than their male counterparts (Ba et al., 2021). Officers also have higher household incomes: on average, officers’ households in our data make over $114,000 a year, whereas a representative group of civilian households would earn roughly $22,000 less.

**FIGURE 3:**
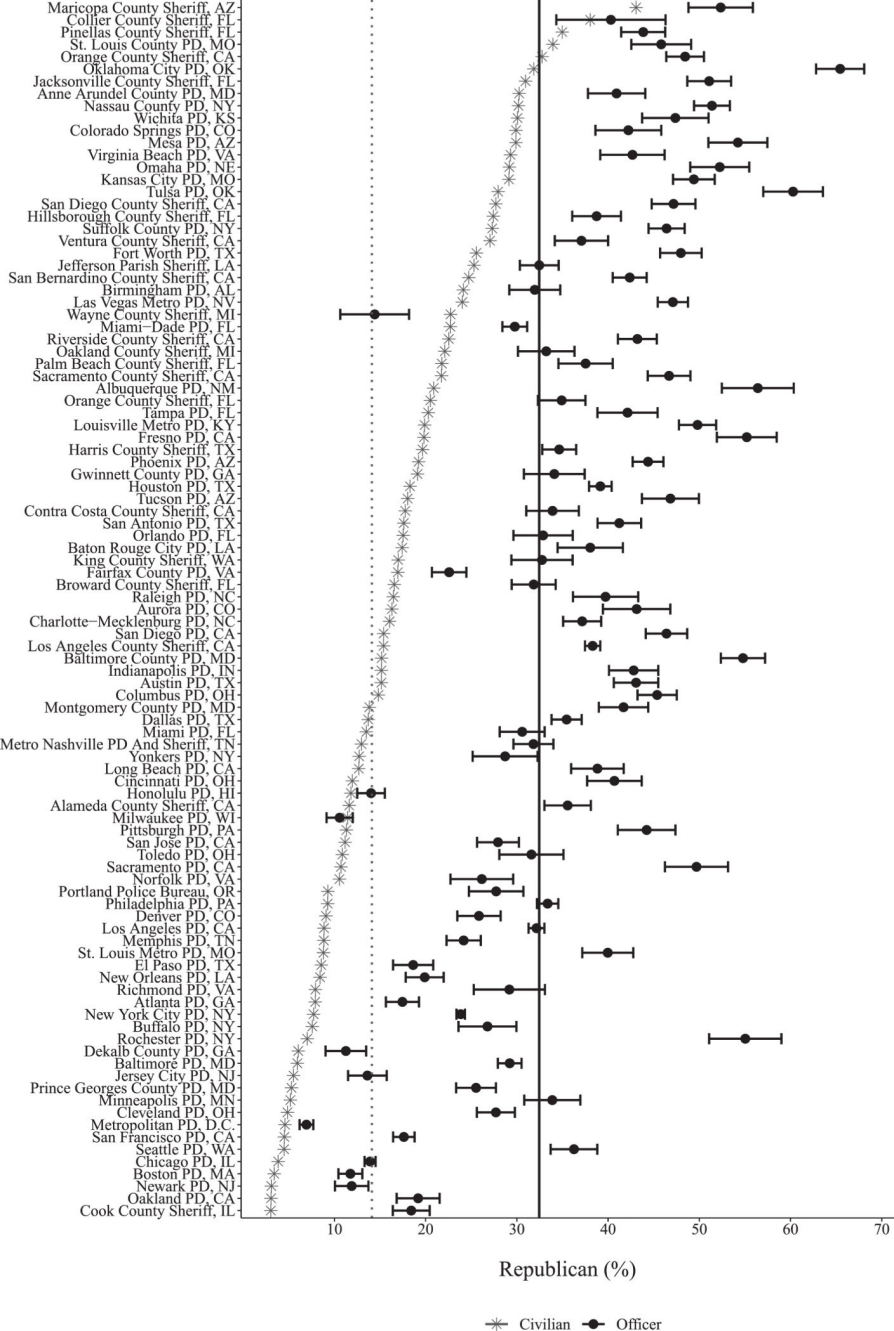
Average shares of Republicans among officers and civilians in the same jurisdictions. *Note*: Black dots are officer shares with 95% confidence intervals. Gray asterisks are civilian Republicans from L2 as a share of voting-age population from Census ACS. The vertical solid black line is the pooled officer mean. The vertical dotted gray line is the hypothetical officer mean if each officer was randomly drawn from their respective jurisdiction.

Our pooled results mask heterogeneity across agencies. To explore this variation, Figure 3 plots each jurisdiction in terms of officer and civilian Republican share; the pooled means from Table 1 are plotted as vertical lines for reference. Agency-level comparisons to civilians on race, voter turnout, gender, age, and household income for all 99 agencies appear in Online Appendix Table F.1 (Appendix pp. 7–9). These results show agencies ranging from unrepresentative and partially representative to highly representative in terms of party identification and race/ethnicity. Representativeness along racial lines does not always correspond to representativeness along partisan lines.

Consider the Rochester, NY, Police Department: a highly unrepresentative jurisdiction in which at least 55% of police officers are Republican, compared to only 10% of residents. In addition, we find that 75% of Rochester officers are White, compared to 38% of civilians. On the other hand, we observe agencies like the L.A. County, CA, Sheriff’s Department, which is highly representative in some racial categories (e.g., 7% Black officers vs. 8% Black residents), but highly unrepresentative politically (38% Republican officers vs. 21% Republican residents). Finally, we also see agencies that are roughly representative on both dimensions, such as the Birmingham, AL, Police Department, comprised of 32% Republican officers (vs. 27% civilians), 37% White officers (vs. 35% civilians), and 61% Black officers (vs. 57% civilians).^17^

## MICRO-LEVEL CASE STUDIES IN CHICAGO AND HOUSTON

We now turn to detailed case studies of two large agencies, the CPD and HPD, where we obtained rich data on officer deployment and enforcement behavior. We use these data to conduct several analyses. First, we assess PR at a more fine-grained level, using deployment data to test whether officers are representative of the civilians with whom they likely interact. Second, we investigate whether officers of different social identities—in particular, political affiliations—treat civilians differently in ways consistent with actively representing partisan preferences for how policing should be conducted. While this analysis would ideally study behavior in even more jurisdictions, we have found that data on day-to-day officer deployment—which is crucial for the credibility of the analysis—is extremely difficult to procure, with many agencies denying open records requests or failing to maintain historical data in usable form. When obtainable, however, deployment records offer a rare opportunity to compare officers while holding working conditions fixed.

### Political representation in police–civilian interactions

**FIGURE 4:**
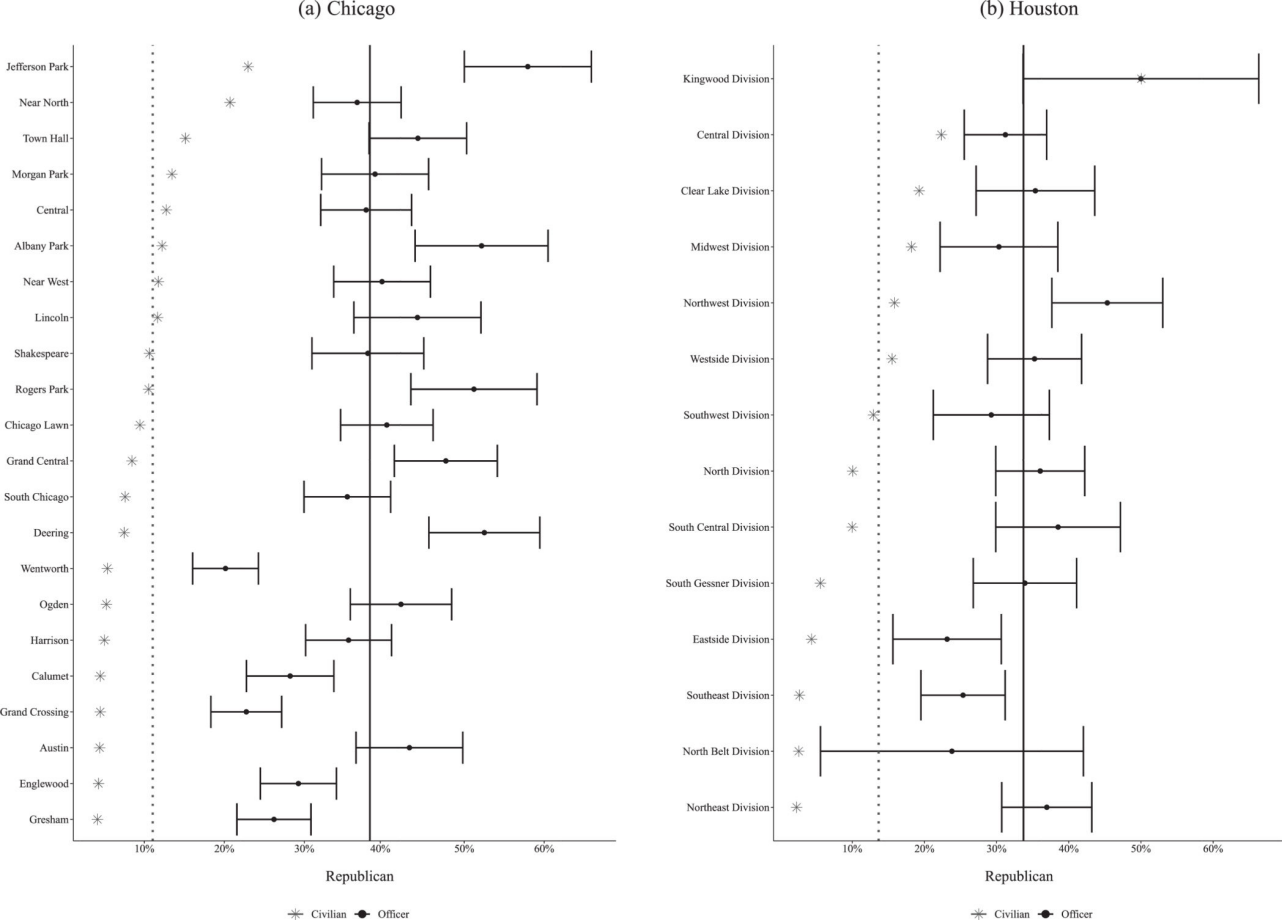
Average shares of Republican officers and civilians in officers’ assigned districts, in Chicago (panel a) and Houston (panel b). *Note*: Black dots are officer shares with 95% confidence intervals. Gray asterisks are civilian Republicans from L2 as a share of voting-age population from Census data. The vertical solid black line is the pooled officer mean. The vertical dotted gray line is the hypothetical officer mean if each officer was randomly drawn from their respective district.

To investigate whether officers are politically representative of the civilians with whom they most likely interact, we associated Chicago and Houston officers with the districts or divisions in which they most frequently worked. We then compared officers to residents of their assigned jurisdictions. Figure 4 shows a striking mismatch for both agencies. In our behavioral data, 15% of CPD officers are Republican. However, even in the district with the highest share of Republican residents, civilians are roughly 9% Republican. And as Figure 4 shows, Republicans are overrepresented among police officers in every Chicago district. We see a similar portrait in Houston. Overall, 36% of HPD officers are Republican. Parity is reached in the most right-leaning division—where approximately half of officers and civilians are Republican—but in every other division, Republican officers are overrepresented. In the division with the lowest share of Republican residents, only 2% of civilians are Republican, compared to 37% of officers.^18^

### A research design to compare officer behavior across partisan groups

We employ a research design developed in Ba et al. (2021) to identify the effect of deploying an officer with one social identity, vs. another officer of a differing identity, to otherwise similar circumstances. From a theoretical perspective, this analysis probes a key observable implication of AR—if officers from different social identities do not treat civilians differently, there is little reason to suspect AR is occurring. We examine the overall volume of stops, arrests, and uses of force made by Democratic (vs. Republican) officers as well as the volume of arrests made for specific types of crimes. We further assess partisan differences in treatment of racial/ethnic minorities. Each behavioral outcome represents one potential channel through which partisan officers might actively represent copartisans’ preferences on how policing should be performed.

To conduct this analysis, we analyze the 2012–2019 CPD shift assignment and enforcement records, collecting new data to double the 2012–2015 coverage of Ba et al. (2021). Our Houston data covers 2017–2020. Tables 2 and 3 summarize these datasets. As the tables show, our data include observations on the behavior of almost 12,000 officers across 6.7 million shifts in Chicago as well as roughly 2,400 officers across 1.2 million shifts in Houston. Appendix Tables E.2 and E.3 show the number of stops, arrests, and uses of force per 100 shifts by officer and civilian group for Chicago and Houston.^19^

We note that the data provided by HPD suffers numerous quality issues, often making judgment calls necessary during preprocessing. For example, (1) officers were not identified by badge or employee numbers in HPD-provided enforcement data, and names were often abbreviated inconsistently even within a single dataset; (2) all instances of the number “8” appear to have been manually deleted from dates and times in the use-of-force data, requiring imputation to remedy; and (3) civilian ethnicity was excluded from stop data despite evidence that HPD tracks this information for its annual reports.

Our analyses compare officers working standard patrol assignments in the same month-year (e.g., January 2012), day of the week, 8-hour shift, and beat (a specific task or assignment, often small patrol areas about one square mile in Chicago). We refer to these units as “MDSBs.” The target quantity in this analysis is the average treatment effect of taking all shifts worked by one group in the MDSB and, counterfactually, reassigning them to officers of another group who were eligible to work in the same MDSB (and vice versa). This quantity is equivalent to the average within-MDSB difference in expected enforcement activity between the two groups of officers. However, these differences cannot be feasibly estimated in MDSBs that have no variation in treatment assignment, for example, when all working officers are Republican. For this reason, we focus on the average treatment effect among MDSBs where comparisons can feasibly be made. We stress that the treatment of interest—the deployment of an officer of one group, vs. another—is inherently bundled. Officers of a particular partisan identity, for example, differ in many ways besides political orientation. In practice, however, commanders can only deploy whole officers; they cannot modify an officer’s identity while holding its correlates fixed, meaning that the bundled treatment effect is in fact the quantity of greatest substantive relevance. Put differently, we seek to estimate the effect of *deploying* an officer of one identity relative to another, with all their associated traits (Sen & Wasow, 2016); we do not seek to estimate the effect of modifying the identity itself.^20^

We use weighted fixed-effects regressions to compare the enforcement decisions of officer groups within each MDSB and aggregate these into an overall estimate of the deployment disparity. Weights based on the within-MDSB prevalence of each group are used to obtain unbiased estimates of the average treatment effect (see Online Appendix D (Appendix pp. 2–3) for additional details on estimation). Standard errors are clustered by an officer. The key assumption underlying this analysis is that, prior to post-deployment decisions about how to spend their shifts, officers from different groups are equally likely to encounter the same types of civilians, scenarios, and conditions within MDSBs. As outlined in Ba et al. (2021), a rotating day-off scheduling system in the CPD greatly limits the ability of officers to select into working environments with systematically different conditions. In line with the assumption of as-if random assignment of officers to shifts within small slices of time and space, balance tests using incident-level crime data show that crime conditions are statistically indistinguishable across officer groups within MDSBs in Chicago (see Online Appendix J, Appendix p. 25).

**FIGURE 5:**
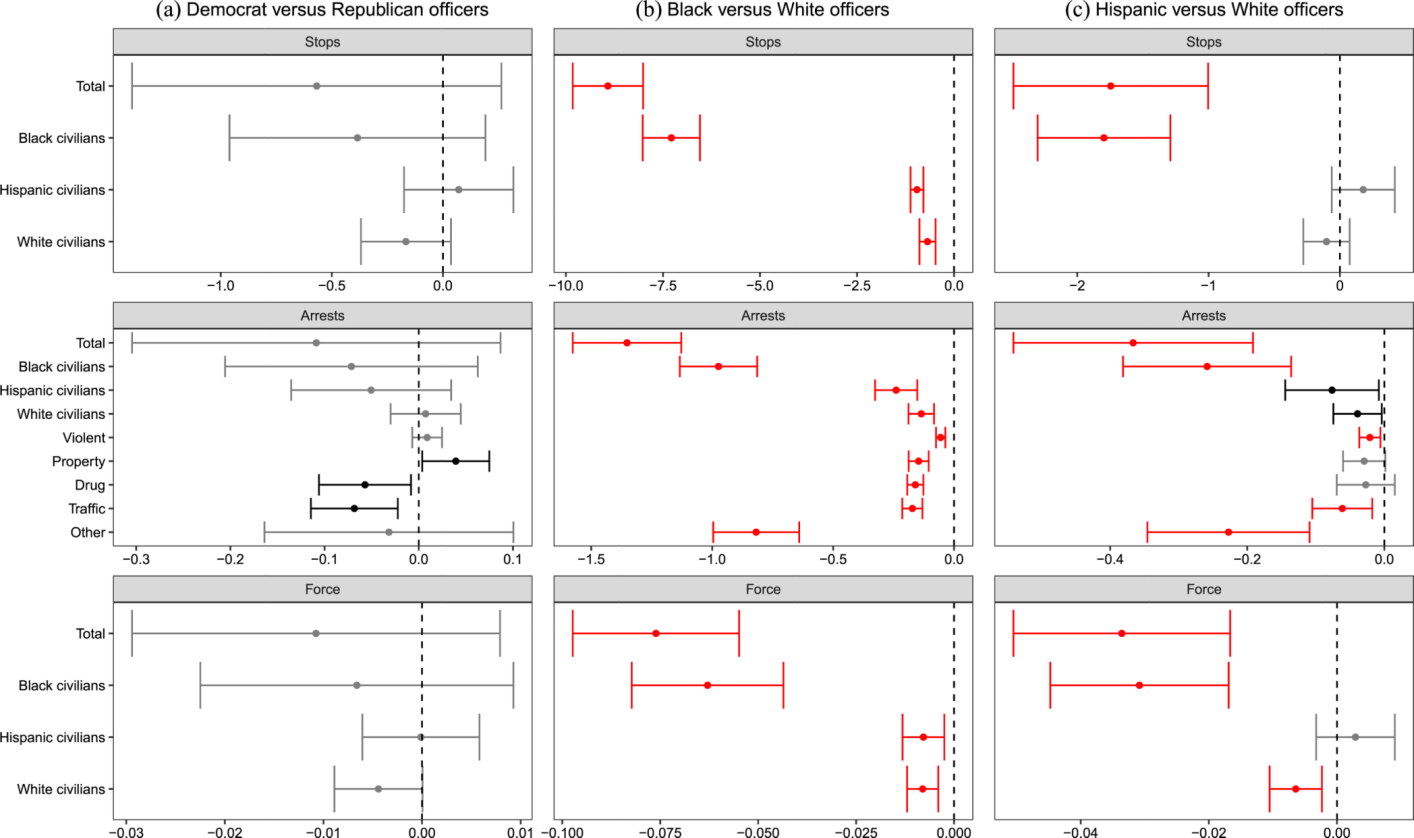
Deployment effects in Chicago. *Note*: The plot displays the effect of deploying a Democrat vs. a Republican officer in similar circumstances on various outcomes. Unadjusted 95% confidence intervas with officer-clustered standard errors displayed. Estimates in gray are nonsignificant. Estimates in black were statistically significant prior to multiple testing corrections. Estimates in red remain significant after multiple testing corrections.

**FIGURE 6:**
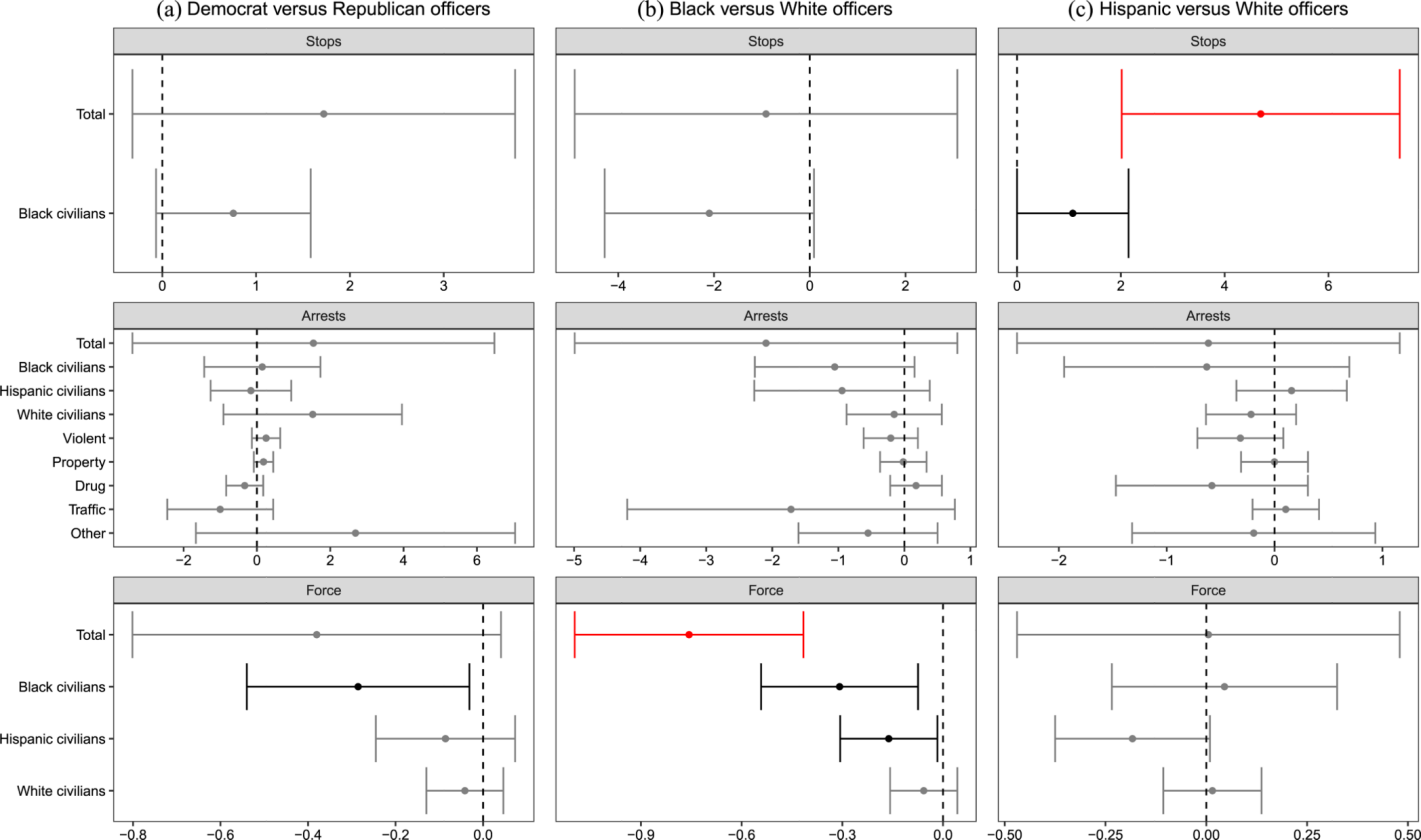
Deployment effects in Houston. *Note*: The plot displays the effect of deploying a Democrat vs. a Republican officerin similar circumstances on various outcomes. Unadjusted 95% confidence intervals with officer-clustered standard errors displayed. Estimates in gray are nonsignificant. Estimates in black were statistically significant prior to multiple testing corrections. Estimates in red remain significant after multiple testing corrections.

**FIGURE 7:**
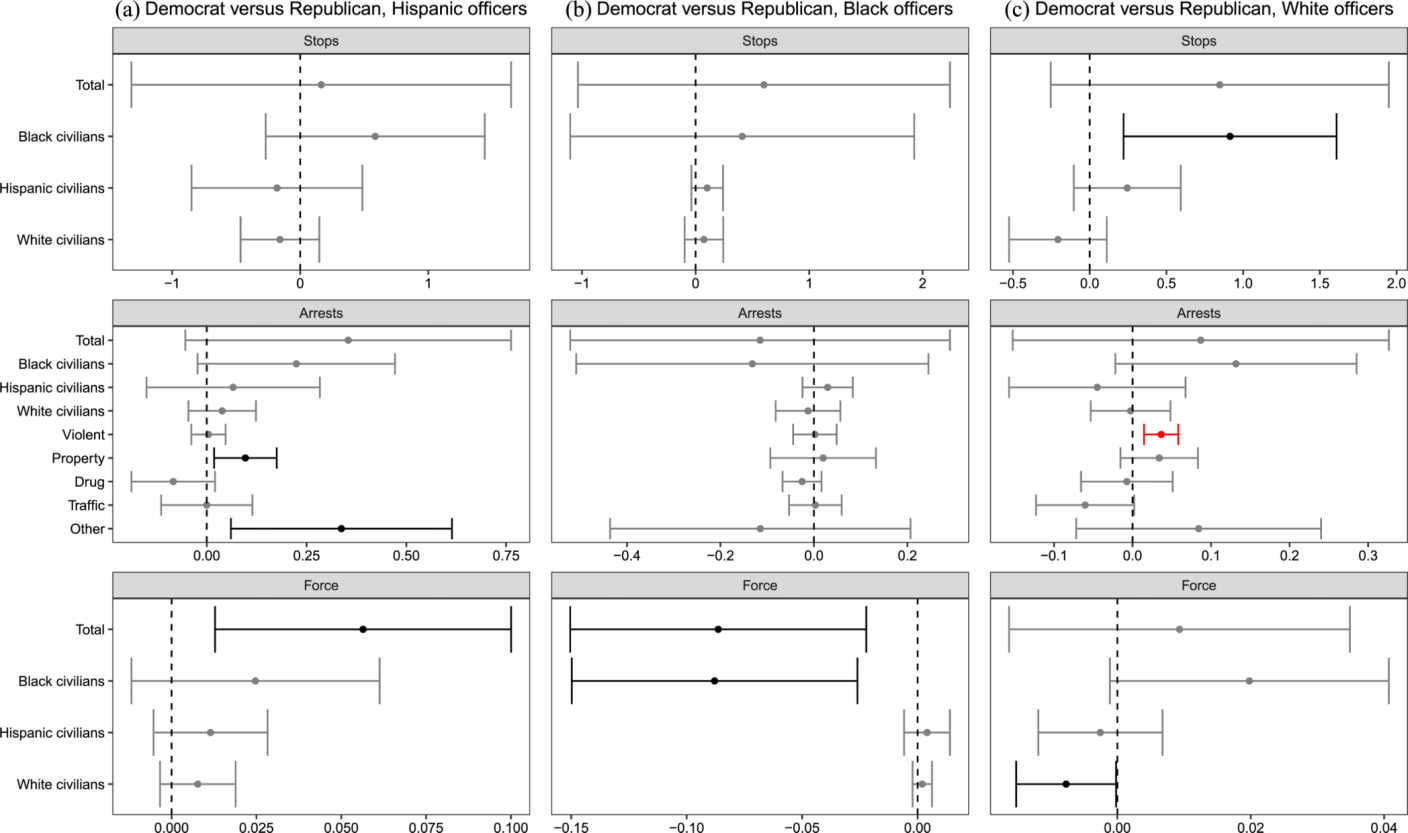
Deployment effects in Chicago within racial groups. *Note*: The plot displays the effect of deploying a Democrat vs. a Republican officer in similar circumstances on various outcomes, separately by racial/ethnic officer group. Unadjusted 95% confidence intervals with officer-clustered standard errors displayed. Estimates in gray are nonsignificant. Estimates in black were statistically significant prior to multiple testing corrections. Estimates in red remain significant after multiple testing corrections.

**FIGURE 8:**
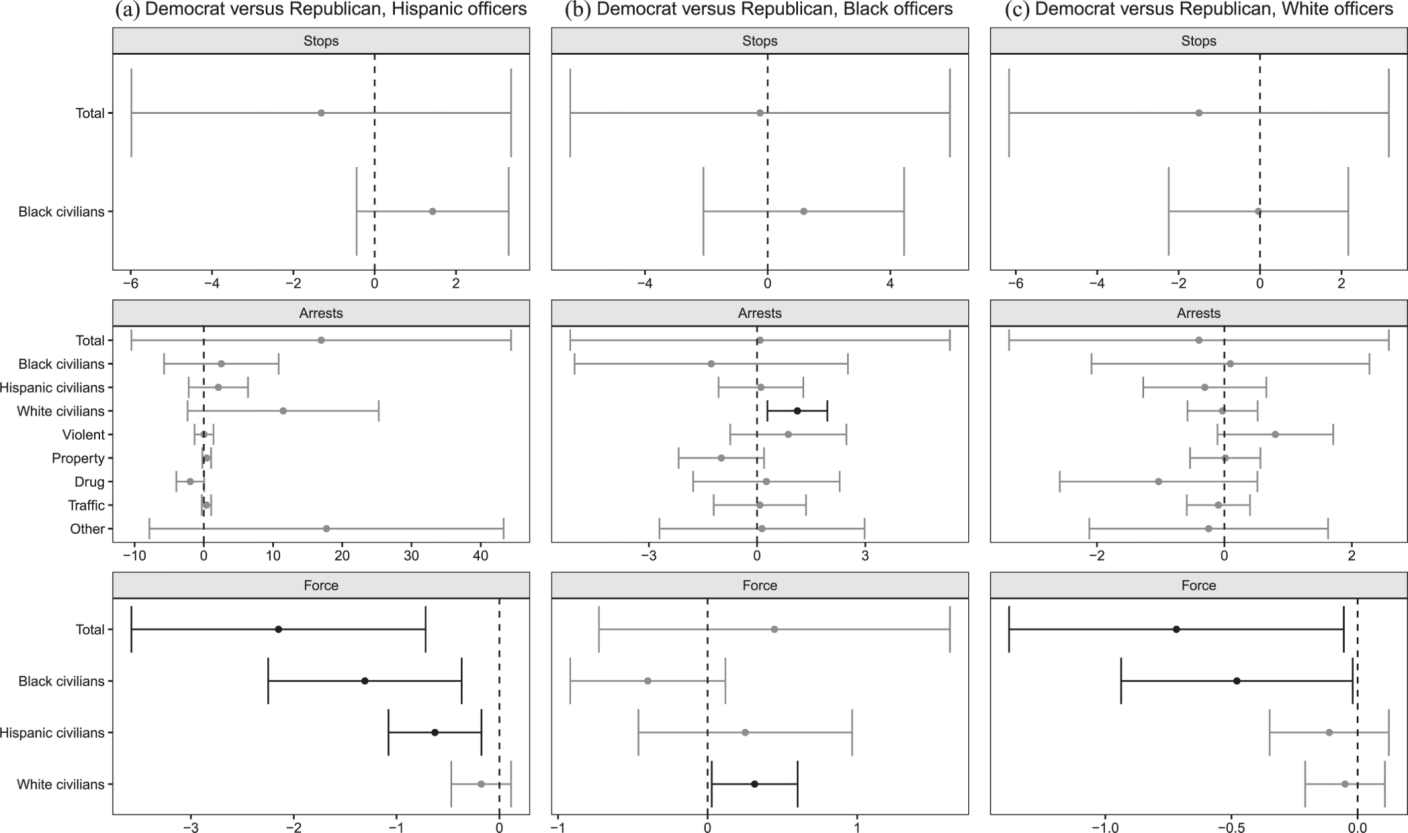
Deployment effects in Houston within racial groups. *Note*: The plot displays the effect of deploying a Democrat vs. a Republican officer in similar circumstances on various outcomes, separately by racial/ethnic officer group. Unadjusted 95% confidence intervals with officer-clustered standard errors displayed. Estimates in gray are nonsignificant. Estimates in black were statistically significant prior to multiple testing corrections. No estimates remain significant after multiple testing corrections.

Our behavioral analyses are organized as follows. At a high level, six comparisons are made. These include unconditional comparisons between (1) Democratic and Republican officers, (2) Black and White officers, and (3) Hispanic and White officers, as well as conditional Democratic–Republican comparisons within (4) Black, (5) Hispanic, and (6) White subsets of officers. These comparisons correspond to six “families” of null hypotheses, each stating that the two officer groups make the same average decisions, across all types of enforcement, when deployed to common circumstances. We note that the effective sample we are analyzing changes across analyses depending on the comparison being made (Aronow & Samii, 2016). Because the MDSBs where comparisons are feasible differ across subsets, it is not possible to compare results across these groups of analyses (e.g., comparing Democratic–Republican differences to Black-White differences) while holding circumstances constant. However, for each of these groups of tests, the logic of the within-MDSB comparisons holds. To account for the large number of analyses performed, we use the hierarchical multiple-testing procedure of Peterson et al. (2016); see Online Appendix D (Appendix pp. 2–3) for details. Note that in Figures 5–8, we depict unadjusted 95% confidence intervals with robust standard errors; results that remain significant after multiple-testing corrections are indicated in red.

### Results of behavioral analysis

We first report our aggregate test of whether Democrats and Republicans behave differently when facing common circumstances (see left panels in Figure 5), which includes all MDSBs where cross-party comparisons can be made. As the figure shows, our unadjusted results suggest that Democrats in Chicago made significantly fewer arrests for drug crimes (0.1 fewer per 100 shifts; *p*_unadj._ = 0.022, *p*_adj._ = 0.344) and traffic crimes (0.1 fewer per 100 shifts; *p*_unadj._ = 0.004, *p*_adj._ = 0.126,), but made more arrests for property crimes (0.04 more per 100 shifts; *p*_unadj._ = 0.030, *p*_adj._ = 0.344). However, these differences lose statistical significance after multiple-testing corrections, as the larger *p*_adj._ values indicate. Similarly, in Houston, Figure 6 shows Democrats used less force against Black civilians than Republicans (0.3 fewer force uses per 100 shifts; *p*_unadj._ = 0.028, *p*_adj._ = 1). Across both cities, after correcting for multiple comparisons, we find no significant differences between Democratic and Republican officers facing common circumstances in terms of total policing activity, activity toward various civilian groups, and arrests for different crime types.

One possible explanation for the lack of detectable differences between Democratic and Republican officers in the aggregate is that these groups are not monolithic. For example, partisan groups contain different proportions of officers with Black, Hispanic, White, or other racial/ethnic identities, and prior work has shown that these other attributes are strongly predictive of officers’ enforcement behavior. In principle, it is possible that this other source of variation could make it statistically difficult to detect partisan differences. To examine this possibility, we next extend our analysis in two ways: (1) by comparing minority to White officers and (2) by comparing Democratic officers to Republican officers *of the same race/ethnicity*.^21^

The central panels in Figure 5 show that across all variants of outcomes, and after correcting for multiple testing, Black officers in Chicago make fewer stops and arrests, and they use force less often, than White officers facing common circumstances. Specifically, Black officers make 8.9 fewer stops, 1.4 fewer arrests, and have 0.1 fewer uses of force per 100 shifts (all *p*_unadj._ ≤ 0.001, *p*_adj._ ≤ 0.001). These reductions are equivalent to 28.1%, 19.4%, and 31.3% of the average output of White officers citywide. Black officers also make 7.3 fewer stops, 1.0 arrests, and 0.06 uses of force involving Black civilians specifically (per 100 shifts; all *p*_unadj._ ≤ 0.001, *p*_adj._ ≤ 0.001). Some of these patterns are shared by Hispanic officers, who make 0.4 fewer arrests overall, 0.3 fewer arrests of Black civilians, 1.7 fewer stops overall, 1.8 fewer stops of Black civilians, and 0.03 fewer uses of force, both overall and against Black civilians specifically (per 100 shifts; all *p*_unadj._ ≤ 0.001, *p*_adj._ ≤ 0.001). Racial/ethnic enforcement differences are less pronounced in Houston, where the HPD’s smaller size, differing deployment patterns, and a number of data issues mean that effects are estimated with substantially more noise.

As Figure 6 shows, Black HPD officers engage in 0.8 fewer uses of force (*p*_unadj._ ≤ 0.001, *p*_adj._ = 0.001) and Hispanic officers engage in 4.7 additional stops per 100 shifts than White officers in comparable circumstances (*p*_unadj._ ≤ 0.001, *p*_adj._ = 0.026), but we do not detect other behavioral differences across racial/ethnic lines. As these two jurisdictions and agencies differ on many dimensions—including racial composition, political history, and local culture—it is difficult to discern why race-based differences are so pronounced in Chicago but less prevalent in Houston. This may be in part due to the aforementioned differences in data quality, but other factors, such as Chicago’s requirement that all officers reside within the city, may also play a role. Future work is necessary to investigate these contextual differences. However, part of the present study’s contribution is to underscore that such variation exists. In a nation of 18,000 law enforcement agencies, discussions of “policing” writ large may often mask important heterogeneity.

Finally, Figure 7 tests whether Democrats in Chicago behave differently, compared to Republican peers of the same race/ethnicity. Prior to multiple-testing corrections, results are mixed: Hispanic Democratic officers appear to use more force than co-ethnic Republicans, whereas Black Democratic officers appear to use less force than co-racial Republicans. As the figure shows, however, only one comparison survives a multiple testing correction, with White Democrats making more violent crime arrests than White Republicans (an increase of 0.04 arrests per 100 shifts; *p*_unadj._ = 0.001, *p*_adj._ = 0.036). In Houston, we find no detectable differences across partisan groups within officer racial/ethnic groups.

## DISCUSSION AND CONCLUSION

Democrats and Republicans strongly disagree on how policing should be conducted in the United States. These sharp divisions motivate a close examination of the partisan affiliations and behavior of a particular group of Americans that is well-situated to translate these preferences into policy: police officers themselves. If officers of different political persuasions hold dramatically different views of how policing should be done, these attitudes may manifest in on-the-job behavior, with potentially severe consequences for civilians.

In this paper, we draw on original data characterizing police officers from 99 of the 100 largest local law enforcement agencies in the United States, as well as micro-level behavioral data in Chicago and Houston, to assess the prevalence and consequences of political diversity in policing. Our results confirm that police differ systematically from the communities they serve in every way we can measure—that is, in the parlance of RB theories, they exhibit deficiencies in PR. The majority of police agencies we study are out of step with the communities they serve, with officers skewing more Republican and being far more politically active. But just as importantly, we find heterogeneity: our broad agency-level data collection allows us to identify some highly representative agencies that could not be discerned in prior, coarser analyses. In addition, we show that representativeness along racial lines does not always correspond to representativeness along partisan lines.

Despite shortfalls of partisan representation in policing, our micro-level analyses using fine-grained Chicago and Houston data also show that officer behavior does not tend to diverge across partisan lines in ways that are statistically detectable. After correcting for multiple comparisons, we find little evidence that Democrats behave differently than Republicans, both in the aggregate and within racial groups. White officers in Chicago represent a notable exception, with White Democrats making more arrests for violent crimes than White Republicans in Chicago.

This stands in stark contrast to the sharp racial/ethnic divides in policing. Consistent with Ba et al. (2021), we find, for example, that Black and Hispanic officers in Chicago make fewer stops and arrests, and they use force less often than White officers facing common circumstances, especially during encounters with Black civilians. In Houston, we find that when facing common circumstances, Black officers use force less often than their White peers, while Hispanic officers make more stops than their White peers. These results paint a complex portrait of how officer identity maps to police–civilian interactions that previous analyses of single jurisdictions and social identities have failed to uncover.

Our paper also offers a template for future data collection efforts for the study of bureaucrats. Unlike other professions such as law and medicine, which provide public-facing lists of accredited members, law enforcement agencies are sometimes reluctant to disclose the identities of public employees. Despite this obstacle, we obtained detailed data on officers from nearly all of the top 100 largest agencies by combining information from open-data portals managed by local governments, data repositories maintained by news agencies and nonprofits, and extensive open records requests—some of which required months of followup communications with municipalities and appeals after initial denials.

Of course, our analysis also has limitations. For one, our data do not allow us to assess whether the deployment of various officer groups has second-order effects on social outcomes such as community trust in police, crime rates, or public safety. However, we view this analysis as a crucial first step in the empirical evaluation of longstanding theories of descriptive representation in the policing context. It also remains exceedingly difficult to obtain the detailed shift assignment records necessary to make principled behavioral comparisons across officer groups. As a result, our behavioral analysis is limited to two major cities. Even when such records can be obtained, months of cleaning and standardization are required before a multijurisdiction analysis is possible. In some cases, such as Houston, consistent officer identifiers are not always available, and extensive manual work is necessary to produce analysis-ready data. The degree to which progress will be made in this literature not only depends on scholars seeking similar administrative data but also on the willingness of agencies to generate, maintain, and distribute high-quality records.

## Supplementary Material

Supplemental materials

Additional supporting information can be found online in the Supporting Information section at the end of this article.

## Figures and Tables

**TABLE 1:** Comparison of average officer and civilian traits.

Variable	Value	Actual officer	Hypothetical representative officer	Difference	*N*

Race	White	51.26%	37.95%	13.31** [13.11, 13.51]	112,446
	Hispanic	23.75	27.98	−4.23** [−4.40, −4.06]	52,089
	Black	16.05	21.26	−5.21** [−5.36, −5.06]	35,207
	Other/unknown race	3.65	3.42	0.23** [0.15, 0.30]	8,000
	Asian	5.30	9.40	−4.10** [−4.19, −4.01]	11,625
Party (voting age population)	Republican	32.45%	14.11%	18.34** [18.15, 18.53]	71,177
	Democratic	31.32	43.50	−12.18** [−12.37, −11.99]	68,705
	Other/unknown party	36.23	42.64	−6.41** [−6.61, −6.20]	79,483
Turnout (voting age population)	General election, 2020	69.39%	54.62%	14.76** [14.57, 14.96]	150,609
Gender	Male	82.75%	48.69%	34.07** [33.91, 34.22]	181,532
	Female	17.25	51.31	−34.07** [−34.22, −33.91]	37,833
Age (years)	—	44.00	36.84	8.07** [8.00, 8.13]	187,382
Household income ($)	—	114,199.67	92,220.54	21,979.13** [21, 704.20, 22, 254.06]	186,778

*Note*: The table displays, from left to right, the attributes of actual officers (means, except where otherwise noted) with a given attribute; the attributes of a hypothetical set of officers randomly drawn from their respective jurisdictions; and the difference between the two. Census data does not provide means or full distributions for age; we therefore report the median of actual officer ages, the median age for a hypothetical set of officers with ages equal to the median age in their jurisdiction, and the difference in means between the two.

** denotes *p* < 0.01;

* denotes *p* < 0.05; brackets contain 95% confidence intervals. *N* indicates the number of officers.

**TABLE 2:** Summary of Chicago data on officer behavior (counts), 2012–2019.

	White	Black	Hispanic	Male	Female	Republican	Democratic	Other/unknown party

Stops	1,037,792	355,786	538,171	1,563,521	368,228	353,242	1,132,438	446,069
Arrests	236,208	84,498	137,462	376,634	81,534	79,299	255,252	123,617
Force	10,512	3,605	5,357	16,777	2,697	3,421	11,004	5,049
Shifts	3,273,026	1,603,495	1,779,986	5,212,874	1,443,633	1,100,840	4,043,087	1,512,580
Officers	5,763	2,682	3,219	8,808	2,856	1,791	6,888	2,985

**TABLE 3:** Summary of Houston data on officer behavior (counts), 2017–2020.

	White	Black	Hispanic	Male	Female	Republican	Democratic	Other/unknown party

Stops	255,280	183,268	206,769	618,884	26,433	316,808	273,192	55,317
Arrests	58,871	27,035	53,591	126,206	13,291	51,296	64,132	24,069
Force	20,773	6,637	15,552	39,278	3,684	16,731	18,618	7,613
Shifts	499,398	297,672	431,422	1,085,435	143,057	462,866	577,503	188,123
Officers	986	553	867	2,088	318	876	1,143	387

## Footnotes

2 In Online Appendix G (Appendix pp. 9–11), we broaden our analysis to examine the neighborhoods in which CPD and HPD officers live, as residency programs are a prominent proposal for integrating officers into local civilian communities (President’s Task Force on 21st Century Policing, 2015). We find the composition of officers’ neighborhoods also differs systematically from that of the city at large. Areas where officers live are similarly higher on shares of Republicans, shares of White residents, voter turnout rates, and household income, compared to jurisdictions overall.

3 The appendix of Donahue (2023) features a “veil of darkness” test comparing the demographics of stopped drivers before and after sunset (Grogger & Ridgeway, 2006), which is robust to selection bias. However, this test has low statistical power and, perhaps relatedly, Donahue (2023) finds no detectable difference in the rates at which Democratic and Republican officers stop Black civilians. In addition, Donahue (2023) matches 68% of officers to the voter file using name and date of birth. Our use of probabilistic record linkage allows us to match more than 85% of officers with at least 90% probability (Enamorado, Fifield, and Imai, 2019).

4 Our analysis uses gradient-boosted decision trees (Chen & Guestrin, 2016) to assess the change in predicted attitudes when a demographic characteristic is included in the model (vs. a baseline model where it is excluded). For each specification of the baseline model (e.g., one that uses only an intercept), the inclusion of the variable (e.g., a model that uses partisan affiliation alongside the intercept) shifts the predicted values by some additive amount. The Shapley value represents the variable’s overall contribution when averaging over all possible baseline model specifications (i.e., inclusion/exclusion decisions for the remaining variables). To estimate this Shapley value, we utilize a version of the computationally efficient SHapley Additive exPlanations (SHAP) method that is tailored for categorical predictors (Amoukou, Brunel, and Tangi, 2022).

5 We began with agencies contained in DOJ (2016), then limited our sample to sheriff’s departments and local or county police. We also excluded state police and sheriff’s departments that do not engage in law enforcement services. Remaining agencies were then ranked by number of full-time sworn officers according to the Census of State and Local Law Enforcement Agencies, the most complete record of agency size available.

6 We were unable to secure data from the Detroit, MI, Police Department.

7 See Online Appendix Table E.1 (Appendix p. 4) for comparisons of officers in our data to (1) officers nationwide and (2) the U.S. population (Hyland & Davis, 2019).

8 After matching officers to voters in the L2 database, we retain all officers with a 0.9 or greater posterior probability of a match. Alternative core results using a cutoff of 0.95 appear in Online Appendix Table I.2 (Appendix p. 19).

9 See Online Appendix A (Appendix p. 1) for details on civilian data. See Online Appendix B (Appendix pp. 1–2) for details on jurisdiction geography and Census merge.

10 For example, Hersh and Goldenberg (2016) used a similar merging approach to obtain physicians’ partisan registration and compared results to a survey of a stratified sample of the matched physicians, which included a question about political ideology. Only 2% reported opposite ideologies to the imputed partisan affiliation.

11 Extreme assumptions about the nature of measurement error—e.g., assuming that an officer is Democratic if even one of their multiple L2 matches fits this description—do affect some conclusions. See extended discussion in Online Appendix I (Appendix pp. 15–24).

12 See Online Appendix Tables H.1 and H.2 for robustness checks related to potential mismeasurement of race/ethnicity.

13 See Online Appendix B (Appendix pp. 1–2) for details on matching tracts to jurisdictions.

14 Note for aggregate results, the total number of observations across racial/ethnic groups differs slightly from the total for other variables. This is the result of rounding after multiplying agency-level proportions from LEMAS (2020) by agency-level officer counts to recover the number of officers in each race/ethnicity category.

15 Specifically, this hypothetical value is computed as $\frac{\sum_{i}X_{i}N_{i}}{\sum_{i}N_{i}}$, where i indexes agencies, ${\bar{X}}_{i}$ refers to the average civilian attribute in the agency’s jurisdiction, and $N_{i}$ is the number of officers employed by the agency.

16 We note that civilian age is computed using data on all civilians, including those too young to serve on police forces, in keeping with our goal of comparing officers to all civilians in their jurisdictions, not just those eligible to serve. However, for reference, the median age among adult civilians is 44. Civilian party identification, computed using voter file records, is restricted to adults. In addition, turnout analyses exclude voter turnout for agencies in Kentucky, which account for about 1% of officers, due to missing data in L2.

17 In response to a reviewer’s comment, we also tested whether disparities differed between police and sheriff’s agencies. As Online Appendix Table G.2 (Appendix p. 12) shows, both types of agencies show overrepresentation of white officers, but the degree of overrepresentation is 5 p.p. larger among police agencies. Likewise, Democrats are underrepresented in both types of agencies, but the underrepresentation is 6 p.p. larger for police agencies. These patterns are consistent with, but not dispositive of, elections promoting descriptive representation in policing.

18 Online Appendix Tables G.3 and G.4 (Appendix pp. 13–14) also display district-level comparisons between officer and civilian race/ethnicity for Chicago and Houston, respectively. Throughout this section, we use officer-level race/ethnicity data provided by CPD and HPD; note that this diverges from the approach in Table 1, which relied on more widely available data sources for consistency. Summary statistics in this section are computed using CPD and HPD data prior to additional filtering described in the next section.

19 We estimate that party affiliations for CPD officers included in this analysis are approximately as follows: White officers: 53% Democrat, 23% Republican; Black officers: 84% Democrat, 5% Republican; Hispanic officers: 49% Democrat, 12% Republican. In Houston, the party affiliations for officers included in the analysis are White officers: 18% Democrat, 63% Republican; Black officers: 66% Democrat, 17% Republican; Hispanic officers: 70% Democrat, 19% Republican.

20 See Hall (2015) for a related discussion on interpreting bundled treatments.

21 We reiterate that even using these refined comparisons, differences in behavior cannot be interpreted as the causal effect of changing an officer’s partisanship: despite holding race/ethnicity fixed, there are numerous other differences between Democratic and Republican officers, such as socioeconomic status. As in our primary analyses, results are best interpreted as the effect on enforcement outcomes that a commander can expect if deploying a randomly drawn officer from one group, vs. another group, among those available to work in a particular place and time.

## References

[R1] Adams IT, McCrain J, Schiff DS, Schiff KJ, and Mourtgos SM. 2024. “Police reform from the top down: Experimental evidence on police executive support for civilian oversight.” Journal of Policy Analysis and Management 51(4): 905–28.

[R2] Amoukou Salim I., Nicolas J-B. Brunel, and Tangi Salaün. 2022. “Accurate Shapley Values for Explaining Tree-based Models.” Paper presented at Proceedings of the 25th International Conference on Artificial Intelligence and Statistics (AISTATS) 2022, Valencia, Spain.

[R3] Aronow Peter M., and Samii Cyrus. 2016. “Does Regression Produce Representative Estimates of Causal Effects?.” American Journal of Political Science 60(1): 250–67.

[R4] Ba Bocar A., Knox Dean, Mummolo Jonathan, and Rivera Roman G.. 2021. “The Role of Officer Race and Gender in Police-Civilian Interactions in Chicago.” Science 371(6530): 696–702. 33574207 10.1126/science.abd8694

[R5] Baumgartner Frank R., Bell Kate, Beyer Luke, Boldrin Tara, Doyle Libby, Govan Lindsey, Halpert Jack, Hicks Jackson, Kyriakoudes Katherine, Lee Cat, 2021. “Intersectional Encounters, Representative Bureaucracy, and the Routine Traffic Stop.” Policy Studies Journal 49(3): 860–86.

[R6] Bendor Jonathan, and Meirowitz Adam. 2004. “Spatial Models of Delegation.” American Political Science Review 98(2): 293–310.

[R7] Chen Tianqi, and Guestrin Carlos. 2016. XGBoost: A Scalable Tree Boosting System. In Proceedings of the 22nd ACM SIGKDD International Conference on Knowledge Discovery and Data Mining (KDD ‘16), 785–794. New York: ACM.

[R8] deGrood Matt. 2023. “HPD’s Use of Force Incidents That Cause Major Injuries Disproportionately Affect Black Residents.” Houston Chronicle. https://www.houstonchronicle.com/news/houston-texas/crime/article/hpd-use-of-force-black-residents-17868850.php.

[R9] DOJ. 2016. “Law Enforcement Agency Roster (LEAR), 2016.” Technical report, U.S. Department of Justice. Office of Justice Programs. Bureau of Justice Statistics. Inter-University Consortium for Political and Social Research [distributor].

[R10] Dolan Julie, and Rosenbloom David H.. 2003. Representative Bureaucracy: Classic Readings and Continuing Controversies. London: Routledge.

[R11] Donahue Samuel Thomas. 2023. “The Politics of Police.” American Sociological Review 88(4): 656–80.

[R12] Eckhouse Laurel. 2019. “Race, Party, and Representation in Criminal Justice Politics.” The Journal of Politics 81(3): 1143–52.

[R13] Enamorado Ted, Fifield Benjamin, and Imai Kosuke. 2017. “fastLink: Fast Probabilistic Record Linkage with Missing Data.”Vienna: The Comprehensive R Archive Network. https://cran.r-project.org/web/packages/fastLink/index.html

[R14] Enamorado Ted, Fifield Benjamin, and Imai Kosuke. 2019. “Using a Probabilistic Model to Assist Merging of Large-scale Administrative Records.” American Political Science Review 113(2):353–71.

[R15] Eversley Melanie. 2017. “Trump Tells Law Enforcement: ‘Don’t be too Nice’ with Suspects.” USA Today. https://www.usatoday.com/story/news/2017/07/29/trump-tells-law-enforcement-dont-too-nice-suspects/522220001/.

[R16] Fraga Bernard L., Holbein John B., and Skovron Christopher. 2018. “Using Nationwide Voter Files to Study the Effects of Election Laws.” Working Paper, University of Virginia.

[R17] Gift Karen, and Gift Thomas. 2015. “Does Politics Influence Hiring? Evidence from a Randomized Experiment.” Political Behavior 37:653–75.

[R18] Goldstein Herman. 1977. Policing a Free Society. Pensacola, FL: Ballinger Publishing.

[R19] Grogger Jeffrey, and Ridgeway Greg. 2006. “Testing for Racial Profiling in Traffic Stops from behind a Veil of Darkness.” Journal of the American Statistical Association 101(475): 878–87.

[R20] Grosjean Pauline, Masera Federico, and Yousaf Hasin. 2023. “Inflammatory Political Campaigns and Racial Bias in Policing.” The Quarterly Journal of Economics 138(1): 413–63.

[R21] Hall Andrew B. 2015. “What Happens When Extremists Win Primaries?.” American Political Science Review 109(1): 18–42.

[R22] Harrell Erika, and Davis Elizabeth. 2020. “Contacts between Police and the Public, 2018–Statistical Tables.” Bureau of Justice Statics Report, NCJ 255730.

[R23] Harris. 2021. “Reimagining Chicago’s Public Safety.” Technical Report, MacArthur Foundation. https://theharrispoll.com/reimagining-chicago-public-safety-macarthur-foundation/

[R24] Hersh Eitan D., and Matthew N Goldenberg. 2016. “Democratic and Republican Physicians Provide Different Care on Politicized Health Issues.” Proceedings of the National Academy of Sciences 113(42): 11811–16. 10.1073/pnas.1606609113 PMC5081578 27698126

[R25] Hoekstra M, and Sloan C. 2020. “Does Race Matter for Police Use of Force? Evidence from 911 Calls.” National Bureau of Economic Research, Working Paper (26774). https://www.aeaweb.org/articles?id=10.1257/aer.20201292

[R26] Huber Gregory A., and Malhotra Neil. 2017. “Political Homophily in Social Relationships: Evidence from Online Dating Behavior.” The Journal of Politics 79(1): 269–83.

[R27] Huber John D., and Shipan Charles R.. 2002. Deliberate Discretion? The Institutional Foundations of Bureaucratic Autonomy. Cambridge, UK: Cambridge University Press.

[R28] Hyland Shelley S., and Davis Elizabeth. 2019. “Local Police Departments, 2016: Personnel.” Washington, DC: Bureau of Justice Statistics (BJS) US Department of Justice, Office of Justice Programs, Bureau of Justice Statistics.

[R29] Iyengar Shanto, and Westwood Sean J.. 2015. “Fear and Loathing Across Party Lines: New Evidence on Group Polarization.” American Journal of Political Science 59(3): 690–707.

[R30] Iyengar Shanto, Lelkes Yphtach, Levendusky Matthew, Malhotra Neil, and Sean J Westwood. 2019. “The Origins and Consequences of Affective Polarization in the United States.” Annual Review of Political Science 22: 129–46.

[R31] Kaplan Jacob. 2023. “Uniform Crime Reporting Program Data: Law Enforcement Officers Killed and Assaulted (LEOKA) 1960–2021.” Ann Arbor, MI: Inter-University Consortium for Political and Social Research.

[R32] Kingsley Donald. 1944. Representative Bureaucracy. Brentwood, CA: Antioch Press.

[R33] Knox Dean, Lucas Christopher, and Wendy K Tam Cho. 2022. “Testing Causal Theories with Learned Proxies.” Annual Review of Political Science 25: 419–41.

[R34] Knox Dean, and Mummolo Jonathan. 2020. “Toward a General Causal Framework for the Study of Racial Bias in Policing.” Journal of Political Institutions and Political Economy 1(3): 341–78.

[R35] Knox Dean, Lowe Will, and Mummolo Jonathan. 2020. “Administrative Records Mask Racially Biased Policing.” American Political Science Review 114(3): 619–37.

[R36] Kringen Anne Li. 2014. “Understanding Barriers That Affect Recruiting and Retaining Female Police Officers: A Mixed Method Approach.” Ph.D. thesis, Texas State University-San Marcos.

[R37] LEMAS. 2020. “Law Enforcement Management and Administrative Statistics.” Technical Report, United States Department of Justice. Office of Justice Programs. Bureau of Justice Statistics. Inter-University Consortium for Political and Social Research.

[R38] Lipsky Michael. 1980. Street-Level Bureaucracy: Dilemmas of the Individual in Public Service. Manhatten, NY: Russell Sage Foundation.

[R39] McConnell Christopher, Margalit Yotam, Malhotra Neil, and Levendusky Matthew. 2018. “The Economic Consequences of Partisanship in a Polarized Era.” American Journal of Political Science 62(1): 5–18.

[R40] McCrary Justin. 2007. “The Effect of Court-ordered Hiring Quotas on the Composition and Quality of Police.” American Economic Review 97(1): 318–53.

[R41] Meier Kenneth J. 2019. “Theoretical Frontiers in Representative Bureaucracy: New Directions for Research.” Perspectives on Public Management and Governance 2(1): 39–56.

[R42] Meier Kenneth J., and Jill Nicholson-Crotty. 2006. “Gender, Representative Bureaucracy, and Law Enforcement: The Case of Sexual Assault.” Public Administration Review 66(6): 850–60.

[R43] Meier Kenneth J., Pennington Michael S., and Warren S Eller. 2005. “Race, Sex, and Clarence Thomas: Representation Change in the EEOC.” Public Administration Review 65(2): 171–79.

[R44] Meier Kenneth John. 1975. “Representative Bureaucracy: An Empirical Analysis.” American Political Science Review 69(2): 526–42.

[R45] Morin Rich, Parker Kim, Stepler Renee, and Mercer Andrew. 2017. Behind the Badge: Amid Protests and Calls for Reform, How Police View Their Jobs, Key Issues and Recent Fatal Encounters between Blacks and Police. Technical Report. Washington, DC: Pew Research Center.

[R46] Parker Kim, and Hurst Kiley. 2021. “Growing Share of Americans Say They Want More Spending on Police in Their Area.” Washington, DC: Pew Research Center. https://www.pewresearch.org/fact-tank/2021/10/26/growing-share-of-americans-say-they-want-more-spending-on-police-in-their-area/.

[R47] Peterson Christine B., Bogomolov Marina, Benjamini Yoav, and Sabatti Chiara. 2016. “Many Phenotypes without Many False Discoveries: Error Controlling Strategies for Multitrait Association Studies.” Genetic Epidemiology 40(1): 45–56. 26626037 10.1002/gepi.21942 PMC4738479

[R48] Pew. 2016. “The American Trends Panel Survey, Wave 20.” Washington, DC: Pew Research Center. https://www.pewresearch.org/politics/dataset/american-trends-panel-wave-20/.

[R49] Pew. 2020. “Majority of Public Favors Giving Civilians the Power to Sue Police Officers for Misconduct.” https://tinyurl.com/yw3b9zry.

[R50] Porter Ethan, and Rogowski Jon C.. 2018. “Partisanship, Bureaucratic Responsiveness, and Election Administration: Evidence from a Field Experiment.” Journal of Public Administration Research and Theory 28(4): 602–17.

[R51] President’s Task Force on 21st Century Policing. 2015. “Final Report of the President’s Task Force on 21st Century Policing.” Technical Report, Office of Community Oriented Policing Services. https://cops.usdoj.gov/pdf/taskforce/taskforce_finalreport.pdf.

[R52] Sen Maya, and Wasow Omar. 2016. “Race as a Bundle of Sticks: Designs that Estimate Effects of Seemingly Immutable Characteristics.” Annual Review of Political Science 19: 499–522.

[R53] Shoub Kelsey, Stauffer Katelyn E., and Song Miyeon. 2021. “Do Female Officers Police Differently? Evidence from Traffic Stops.” American Journal of Political Science 65(3): 755–69.

[R54] Sklansky David Alan. 2005. “Not Your Father’s Police Department: Making Sense of the New Demographics of Law Enforcement.” The Journal of Criminal Law and Criminology 96: 1209–44.

[R55] Frank Stephenson E. 2011. “Strategic Voting in Open Primaries: Evidence from Rush Limbaugh’s ‘Operation Chaos’.” Public Choice 148(3): 445–57.

[R56] Thompson Daniel M. 2020. “How Partisan Is Local Law Enforcement? Evidence from Sheriff Cooperation with Immigration Authorities.” American Political Science Review 114(1): 222–36.

[R57] Vasquez Lucio. 2023. “Report Finds Racial Disparities Among Houstonians Pulled Over for Non-Moving Traffic Violations.” Houston Public Media. https://shorturl.at/dFHPY.

[R58] West Jeremy. 2018. “Racial Bias in Police Investigations.” Working Paper. https://people.ucsc.edu/∼jwest1/articles/West_RacialBiasPolice.pdf

[R59] Westwood Sean J., and Peterson Erik. 2022. “The Inseparability of Race and Partisanship in the United States.” Political Behavior 44: 1125–47.

[R60] Wilkins Vicky M., and Brian N Williams. 2008. “Black or Blue: Racial Profiling and Representative Bureaucracy.” Public Administration Review 68(4): 654–64.

[R61] Wilson James Q. 1968. Varieties of Police Behavior. Cambridge, MA: Harvard University Press.

