# Supplementary material for: Political diversity in U.S. police agencies
Source: Am J Pol Sci. Author manuscript; Available in PMC 2025 Aug 28. (PMC12383486; doi:10.1111/ajps.12945)
Supplement: Supplemental materials [file NIHMS2077287-supplement-Supplemental_materials.pdf]

Online Appendix

Table of Contents

A Civilian comparison data 1

B Voter File Record Linkage 1

C Data on Officer Race/Ethnicity and Gender 2

D Estimation of Behavioral Differences 2

E Descriptive Statistics 3

F Within-Jurisdiction Comparisons 7

G Officers' Place of Residence 9

H Measurement Error in Race/Ethnicity 15

I Measurement Error in Party ID 15

J Balance Tests for Behavioral Analysis in Chicago 25

A Civilian comparison data

We compare officers to civilians who live in their agency's jurisdiction. For individual-level data on officers and civilians registered to vote, data come from L2. These data contains the same variables as those used for officers: political party, race/ethnicity, gender, age, and household income. For data on all residents of the jurisdiction we use the American Community Survey (ACS) 2015–2019 data.[20]

B Voter File Record Linkage

To obtain officer-level data, we matched each officer to L2 records for individuals living in the agency's county and any neighboring counties, since officers may commute from outside the jurisdiction. For civilian data, however, we only include people who live within the jurisdiction of each agency. We define a jurisdiction as the area for which each agency claims primary responsibility. More specifically, the area is the county or Census Place (typically a city) where the agency claims authority. In the case of city police departments, this is the city itself. The jurisdiction for the Philadelphia Police Department, for example, is the census place called the City of Philadelphia. For sheriffs' offices, we use self-described jurisdictions per official websites. For example, Wayne County Sheriff's Office in Michigan defines their jurisdiction as "unincorporated villages and townships within Wayne County," [21] meaning that incorporated places in the county—such as Detroit, the seat of Wayne County—are not included. Sheriffs' offices variously cover only unincorporated places in a county, specific parts of the county including both incorporated and unincorporated places, or all of a county.

For both L2- and Census-based comparison groups, we used all people who reside in a Census tract within the agency's jurisdiction. A Census tract is a small geographic unit that covers an average of 4,000 people and in urban areas is the Census' rough approximation of a neighborhood.[22] Census tracts are fully contained within counties, but can extend to cover multiple Census Places (e.g. cities, towns) meaning that different parts of a single tract may lie inside and outside of an agency's jurisdiction. This is rare and occurs primarily in extremely rural areas with low population density.

Each individual in L2 data is associated with an address (including tract, county and state). For computational efficiency, we operate at the tract level when processing L2 data. Tracts with fewer than 100 entries in L2 were excluded. We spatially join the remaining L2 tracts with Census Place shapefiles from the US Census. Tracts that were not in any Place were considered to be in an unincorporated part of that county. We then used the jurisdiction for each agency, as defined above, to identify all tracts for which an agency has at least partial jurisdiction. For example, an agency whose jurisdiction is only a single Census Place (e.g. City of Philadelphia) was assigned every tract in that Place. An agency whose jurisdiction is an entire county, excluding certain Places, was assigned all tracts in that county other than those in the excluded Places. We used the same tract-based operationalization of jurisdiction when analyzing both L2 and Census data.

In the case of officers matching to multiple L2 records, the record with the highest match probability is retained. If there are multiple records that are tied for highest match probability, one is randomly selected.

[20]While the 2020 decennial Census is complete, currently available data does not contain all of the variables that we use.

[21]https://waynecountysheriff.com/about/

[22]https://www2.census.gov/geo/pdfs/reference/GARM/Ch1GARM.pdf

We note that approximately 38% of officers had more than one match after retaining only matches with the highest match probability. The median number of matches was one. Of officers with more than one match, 30% had two matches, 14% had three matches, 8% had four matches, 6% had five matches, 4% had six matches, 3% had seven matches, 3% had eight matches, 2% had nine matches, and the remaining 29% had 10 or more matches.

See Appendix sections H (Appendix p. 14) and I (Appendix pp. 14-23) for a series of robustness checks gauging the impact of potential mismatches.

C Data on Officer Race/Ethnicity and Gender

As explained in the main text, we rely on 2021 LEOKA data (Kaplan, 2023) for gender data on agencies, due to its near-complete coverage. When agencies do not report officer gender in 2021 we use their submissions from either 2020 or 2019. Seven agencies did not report in 2021, but did report in 2020; two agencies did not report in either 2021 or 2020, but did report in 2019. The seven agencies that use 2020 data are: Chicago Police Department, Cincinnati Police Department, Columbus Police Department, Indianapolis Police, Jacksonville City County Police Department, Nassau County Police Department, and Philadelphia Police Department. We use data from 2019 for Wichita Police Department and for New Orleans Police Department. In addition, because LEOKA data does not contain racial/ethnic measures, we obtain those from the 2020 LEMAS data for 86% of agencies, and use L2 estimates of officers' racial and ethnic identities for the remaining agencies.

D Estimation of Behavioral Differences

Our estimation strategy is based on an extension of Ba et al. (2021), which computes average differences in counts of police behaviors using OLS regressions on MDSB-demeaned data, a computationally efficient procedure that is equivalent to fixed-effects regression when combined with our degrees-of-freedom correction to account the demeaning step. We report 95% confidence intervals that cluster on officers, ensuring that inferences are robust to arbitrary within-officer dependence, including overwork in one shift causing less effort in the following shift, life events causing fluctuation in officer behavior on a timescale of a few months, or discontinuous life events e.g. birth of a child causing long-term changes in behavior. We weight each observation inversely by the variance of officer group membership in the MDSB to which it belongs, ensuring that regressions return unbiased estimates of the average treatment effect. Note: in Chicago, beat codes do not always denote geographic locations. However, additional qualitative information on these codes indicates that officers assigned to the same beat code are working in common circumstances, even though their precise location is sometimes unknown. See Section S1.6 of the Supplementary Information in Ba et al. (2021) for an extended discussion of this issue.

At a high level, six comparisons are made. These include unconditional comparisons between (1) Democratic and Republican officers, (2) Black and White officers, and (3) Hispanic and White officers, as well as conditional Democratic-Republican comparisons within (4) Black, (5) Hispanic, and (6) White subsets of officers. These comparisons correspond to six "families" of null hypotheses, each stating that the two officer groups make the same average decisions, across all types of enforcement, when deployed to common circumstances.

Within each family of hypothesis tests, we test an average of 16 hypotheses about specific forms of enforcement—relating to the numbers of stops, arrests, and uses of force involving various civilian demographic groups and crime types.[23]

To correct for multiple comparisons, we use the hierarchical multiple-testing procedure of Peterson et al. (2016). High-level p-values are obtained with a two-step method: (1) Simes' method (Simes, 1986) is used to test whether all specific tests within a family are jointly null; and (2) a Benjamini-Hochberg (BH) correction (Benjamini and Hochberg, 1995) is used to correct for the fact that there are six family-level tests. Low-level p-values are calculated with a complementary two-step method: (1) BH corrections are applied to the raw p-values, and (2) these values are further inflated based on the proportion of families that are insignificant.

E Descriptive Statistics

[23]In Chicago, we examine 17 outcomes. Of these, 12 represent the number of stops, arrests, and uses of force involving all civilians as well as Black, Hispanic, and White civilians specifically. Additional outcomes capture arrests for drug, property, traffic, violent, and other crimes. In Houston, we examine 15 outcomes due to the absence of ethnicity information, which makes it impossible to distinguish Hispanic and White civilians in stops.

Table E.1: **Descriptive Statistics on Police Officers.** Demographics of police officers in our sample relative to police nationwide and the U.S. as a whole. In-sample estimates for police offices from various sources (see Data and Measurement section). National police estimates from Hyland and Davis (2019). National party identification estimates from 2020 American National Election Studies; partisan leaners counted as independents. Other national estimates from U.S. Census. These statistics show our officers skew heavily male (83%) and have much higher household income than the average American household ($114,200 vs. $62,843, respectively). Officers in our data are more racially and ethnically diverse than both officers nationwide and the U.S. population, likely due to our focus on large population centers, which tend to be themselves diverse. Still, the jurisdictions we study—covering 26.7% of the U.S. population and responsible for investigating 41.6% of all murders and conducting 17.4% of all arrests reported to the FBI in 2019 (Kaplan, 2020, 2022)—are important to study in their own right. To generate these numbers we take the sum of murders and arrests, respectively, for the studied agencies, divided by the number of murders and arrests reported by all agencies in 2019.

Variable	Values	Officers in sample	Police in U.S.	U.S.
Race	White	51.26	71.5	60.70
	Hispanic	23.75	12.5	18.00
	Black	16.05	11.4	12.30
	Other/unknown	3.65	4.7	3.60
	Asian	5.30	–	5.50
Party	Republican	32.45	–	31.54
	Democratic	31.32	–	34.72
	Other/unknown party	36.23	–	33.74
Gender	Male	82.75	87.7	49.20
	Female	17.25	12.3	50.80
Age (years)	–	44.00	–	38.10
Household income ($)	–	114,199.70	–	62,843.00
N		219,365.00	701,000.0	330 mm

Table E.2: **Chicago stops, arrests, and uses of force per 100 shifts, by officer and civilian group.**

Officer group	White	Hispanic	Black	Male	Female	Rep.	Dem.	Other party
Black civ.	19.43	17.53	18.31	19.23	16.56	18.45	18.47	19.28
White civ.	4.65	3.60	1.80	3.74	3.49	4.91	3.50	3.29
Hispanic civ.	6.23	7.86	1.39	5.83	4.30	7.04	4.96	5.83
Total civ.	31.71	30.23	22.19	29.99	25.51	32.09	28.01	29.49

(a) **Stops per 100 shifts, by officer and civilian group.**

Officer group	White	Hispanic	Black	Male	Female	Rep.	Dem.	Other party
Black civ.	4.65	4.96	4.54	4.92	3.92	4.46	4.44	5.58
White civ.	0.88	0.79	0.30	0.74	0.63	0.88	0.64	0.82
Hispanic civ.	1.61	1.90	0.39	1.49	1.04	1.78	1.17	1.71
Total civ.	7.22	7.72	5.27	7.23	5.65	7.20	6.31	8.17

(b) **Arrests per 100 shifts, by officer and civilian group.**

Officer group	White	Hispanic	Black	Male	Female	Rep.	Dem.	Other party
Black civ.	0.23	0.21	0.19	0.24	0.13	0.21	0.20	0.25
White civ.	0.03	0.02	0.01	0.03	0.02	0.03	0.02	0.03
Hispanic civ.	0.05	0.05	0.01	0.04	0.02	0.05	0.03	0.05
Total civ.	0.32	0.30	0.22	0.32	0.19	0.31	0.27	0.33

(c) **Uses of force per 100 shifts, by officer and civilian group.**

Table E.3: **Houston stops, arrests, and uses of force per 100 shifts, by officer and civilian group.**

Officer group	White	Hispanic	Black	Male	Female	Rep.	Dem.	Other party
Black civ.	13.32	18.63	13.28	15.71	6.11	17.98	13.70	9.01
Total civ.	51.12	61.57	47.93	57.02	18.48	68.44	47.31	29.40

(a) **Stops per 100 shifts, by officer and civilian group.**

Officer group	White	Hispanic	Black	Male	Female	Rep.	Dem.	Other party
Black civ.	6.16	5.32	6.08	6.08	4.78	5.73	5.81	6.77
White civ.	1.58	1.19	1.72	1.57	1.28	1.54	1.47	1.70
Hispanic civ.	2.79	1.73	3.34	2.79	2.22	2.62	2.72	3.02
Total civ.	11.79	9.08	12.42	11.63	9.29	11.08	11.11	12.79

(b) **Arrests per 100 shifts, by officer and civilian group.**

Officer group	White	Hispanic	Black	Male	Female	Rep.	Dem.	Other party
Black civ.	1.88	1.15	1.62	1.66	1.26	1.63	1.50	1.90
White civ.	0.46	0.28	0.38	0.40	0.32	0.41	0.34	0.46
Hispanic civ.	1.15	0.48	1.06	0.99	0.66	0.96	0.89	1.14
Total civ.	4.16	2.23	3.60	3.62	2.58	3.61	3.22	4.05

(c) **Uses of force per 100 shifts, by officer and civilian group.**

F Within-Jurisdiction Comparisons

Agency		White (%)	Hispanic (%)	Black (%)	Other/ unknown race (%)	Asian (%)	Democratic (%)	Republican (%)	Other/ unknown party (%)	Turnout (voting age population)	Male (%)	Female (%)	Age (years)	Household income ($)
Alameda County Sheriff, CA	Officers	59.06*	15.61*	10.16*	4.20*	10.97*	27.91*	35.57*	36.52*	72.90*	87.33*	12.67*	45.00*	148,576.62*
	Civilians	31.50	24.40	7.90	5.70	30.50	52.80	15.70	31.60	82.60	49.40	50.60	39.53	142,168.74
Albuquerque PD, NM	Officers	52.39*	39.47*	0.00*	7.61	0.53*	19.90*	56.41*	23.68	81.91	86.02*	13.98*	42.00*	101,322.84*
	Civilians	38.80	49.50	2.60	6.40	2.70	48.40	28.10	23.50	79.90	48.90	51.10	37.93	74,444.38
Anne Aroundel County PD, MD	Officers	80.38*	3.08*	14.21	0.53*	1.80*	21.74*	40.93*	37.33*	62.25*	85.68*	14.32*	39.00	133,895.69*
	Civilians	68.70	7.80	15.80	3.90	3.80	43.00	33.10	23.90	76.30	49.10	50.90	39.62	125,186.26
Atlanta PD, GA	Officers	29.33*	5.11	63.24*	0.77*	1.54*	52.43*	17.46*	30.11*	63.36	81.95*	18.05*	43.00*	101,074.31
	Civilians	37.60	4.20	51.50	2.40	4.30	73.30	8.20	18.50	62.20	48.20	51.80	34.78	102,188.66
Aurora PD, CO	Officers	79.16*	10.42*	3.76*	4.49	2.17*	8.54*	43.13*	48.34*	78.00*	88.71*	11.29*	42.00*	128,488.70*
	Civilians	46.70	26.90	14.90	4.60	6.80	36.10	20.90	43.00	83.00	49.70	50.30	35.37	89,350.88
Austin PD, TX	Officers	66.60*	21.78*	7.60	1.51*	2.51*	31.14*	43.06*	25.80*	72.82*	89.01*	10.99*	45.00*	118,422.88*
	Civilians	49.10	33.40	7.40	2.80	7.30	61.30	19.60	19.10	76.50	50.50	49.50	34.89	106,135.19
Baltimore County PD, MD	Officers	79.56*	2.35*	15.26*	0.68*	2.16*	21.87*	54.79*	23.35*	76.90*	82.77*	17.23*	41.50*	121,241.00*
	Civilians	44.70	5.40	42.60	2.90	4.50	64.60	17.90	17.50	67.60	47.20	52.80	38.69	90,048.91
Baltimore PD, MD	Officers	44.40*	12.53*	40.53*	0.28*	2.26	36.04*	29.21*	34.74*	60.77	84.19*	15.81*	46.00*	112,276.43*
	Civilians	27.60	5.40	61.60	2.90	2.50	77.90	7.40	14.70	60.70	47.00	53.00	36.48	73,579.96
Baton Rouge City PD, LA	Officers	60.62*	1.84*	36.40*	0.00*	1.13*	34.65*	38.05*	27.30	81.90*	90.10*	9.90*	43.00*	99,406.15*
	Civilians	38.70	4.40	51.50	1.90	3.40	51.80	22.40	25.80	69.00	47.80	52.20	33.61	71,381.90
Birmingham PD, AL	Officers	37.03	0.19*	60.54*	1.87	0.37*	62.15*	31.96*	5.89*	74.86*	86.07*	13.93*	44.00*	82,515.96*
	Civilians	35.40	4.00	57.30	1.70	1.60	69.40	27.30	3.30	65.90	47.10	52.90	37.53	72,188.57
Boston PD, MA	Officers	69.89*	11.08*	10.48*	6.39*	2.16*	26.72*	11.75*	61.54*	76.54*	85.76*	14.24*	49.00*	136,974.91*
	Civilians	44.50	19.80	22.70	3.40	9.60	49.20	5.00	45.80	72.60	48.00	52.00	33.54	100,987.60
Broward County Sheriff, FL	Officers	48.88*	26.61	21.16*	1.40*	1.96*	28.42*	31.84*	39.73*	69.41*	86.80*	13.20*	43.00*	108,136.03*
	Civilians	36.60	27.30	30.10	2.70	3.30	51.00	20.40	28.60	74.50	49.00	51.00	40.86	85,697.49
Buffalo PD, NY	Officers	67.47*	8.97*	21.29*	1.74*	0.54*	44.44*	26.77*	28.78*	76.97*	80.19*	19.81*	47.00*	96,956.41*
	Civilians	43.10	12.30	35.60	3.30	5.80	67.60	9.20	23.20	61.40	47.70	52.30	34.13	54,432.29
Charlotte-Mecklenburg PD, NC	Officers	68.63*	6.06*	16.42*	7.24*	1.65*	16.44*	37.16*	46.40*	71.89*	85.17*	14.83*	40.00*	106,445.15*
	Civilians	42.30	14.10	34.00	3.20	6.30	46.40	18.90	34.70	77.00	48.00	52.00	35.28	93,640.73
Chicago PD, IL	Officers	47.01*	28.08	20.23*	1.25*	3.44*	55.22*	13.91*	30.87*	76.68*	76.77*	23.23*	44.00*	106,716.58*
	Civilians	33.50	28.70	29.10	2.20	6.50	67.20	4.80	28.00	65.60	48.60	51.40	35.52	86,285.44
Cincinnati PD, OH	Officers	68.27*	0.19*	28.27*	3.17	0.10*	22.85*	40.69*	36.45*	73.67*	76.95*	23.05*	48.00*	109,367.49*
	Civilians	51.00	3.80	39.40	3.70	2.10	55.90	14.00	30.10	70.20	48.40	51.60	34.01	65,613.80
Cleveland PD, OH	Officers	66.89*	9.21*	22.43*	1.41*	0.06*	32.11*	27.70*	40.19*	73.32*	82.65*	17.35*	48.00*	85,443.25*
	Civilians	33.70	11.90	48.30	3.60	2.50	63.50	6.20	30.30	58.40	48.10	51.90	37.17	45,996.85
Collier County Sheriff, FL	Officers	80.23*	14.34*	3.10*	1.94	0.39*	9.69*	40.31*	50.00*	51.94*	85.66*	14.34*	40.50*	102,648.70
	Civilians	62.80	27.90	6.70	1.30	1.30	24.60	49.30	26.10	83.90	49.30	50.70	50.30	105,857.78
Colorado Springs PD, CO	Officers	82.36*	10.28*	4.17*	0.42*	2.78	8.89*	42.22*	48.89*	75.00*	83.33*	16.67*	42.00*	115,307.85*
	Civilians	69.90	16.90	5.70	4.80	2.80	21.40	35.20	43.40	84.80	50.10	49.90	36.37	88,822.61
Columbus PD, OH	Officers	86.71*	1.61*	9.64*	0.97*	1.07*	17.04*	45.37*	37.59	81.89*	88.80*	11.20*	49.00*	117,215.03*
	Civilians	59.20	5.80	25.10	4.10	5.70	42.30	18.30	39.40	74.20	48.90	51.10	34.32	76,750.07
Contra Costa County Sheriff, CA	Officers	63.10*	18.56	6.38	8.20*	3.76*	28.31*	33.91*	37.78*	74.20*	85.02*	14.98*	44.00*	140,269.15*
	Civilians	53.50	20.10	5.00	5.30	16.00	50.40	21.30	28.30	86.30	49.10	50.90	42.14	168,748.68
Cook County Sheriff, IL	Officers	38.20*	24.81*	35.36*	0.35	1.28*	52.02*	18.43*	29.55*	71.30*	73.21*	26.79*	50.00*	103,477.31*
	Civilians	15.80	83.60	0.00	0.60	0.00	25.60	6.90	67.50	46.90	47.80	52.20	24.50	46,678.44
Dallas PD, TX	Officers	44.95*	25.52*	25.77*	0.90*	2.86*	33.31*	35.45*	31.23*	63.83*	80.97*	19.03*	46.00*	114,976.13*
	Civilians	29.30	41.00	23.70	2.00	4.00	66.10	21.30	12.60	68.50	49.50	50.50	33.41	81,583.54
Dekalb County PD, GA	Officers	29.24*	4.79	63.78*	0.65*	1.55*	55.76*	11.25*	32.99*	56.92*	79.43*	20.57*	41.00*	89,409.53*
	Civilians	20.70	5.10	67.50	2.60	4.10	81.80	6.30	11.90	65.80	46.30	53.70	37.17	79,784.37
Denver PD, CO	Officers	64.34*	21.86*	8.90	2.15*	2.76	21.47*	25.84*	52.68*	70.48*	85.20*	14.80*	48.00*	119,848.55*
	Civilians	54.20	29.90	8.90	3.40	3.60	47.00	11.40	41.60	86.40	50.10	49.90	35.09	98,085.25
El Paso PD, TX	Officers	14.98*	81.45	2.25*	0.17*	1.16	70.80*	18.64*	10.57*	64.23*	86.02*	13.98*	42.00*	74,383.85*
	Civilians	12.50	81.80	3.10	1.30	1.20	82.90	11.50	5.60	58.60	49.00	51.00	33.88	64,323.75
Fairfax County PD, VA	Officers	79.60*	7.84*	1.06*	6.20*	5.30*	31.53*	22.58*	45.89*	60.20*	82.94*	17.06*	41.00*	155,032.44*
	Civilians	50.80	16.00	9.60	4.30	19.30	64.40	20.30	15.30	80.00	49.50	50.50	39.17	159,196.16
Fort Worth PD, TX	Officers	68.25*	18.11*	6.33*	6.15*	1.16*	31.05*	47.98*	20.97*	73.15*	86.79*	13.21*	46.00*	110,367.84*
	Civilians	41.80	33.70	17.40	2.70	4.30	47.60	34.70	17.70	69.60	48.90	51.10	33.21	84,488.63
Fresno PD, CA	Officers	48.30*	38.80*	5.88	0.34*	6.67*	18.55*	55.20*	26.24*	77.15*	88.46*	11.54*	42.00*	113,003.66*
	Civilians	28.00	49.20	6.60	2.90	13.30	42.60	28.10	29.40	71.60	49.20	50.80	32.41	70,003.28
Gwinnett County PD, GA	Officers	75.03*	7.46*	14.67*	0.77*	2.06*	21.49*	34.11*	44.40*	69.88	89.96*	10.04*	37.00*	97,447.43
	Civilians	39.50	21.60	25.00	2.80	11.10	41.50	22.40	36.00	71.40	48.90	51.10	35.72	94,655.81
Harris County Sheriff, TX	Officers	31.63*	32.58*	32.18*	0.32*	3.29*	43.02*	34.64*	22.34*	68.61	82.70*	17.30*	47.00*	103,529.01*
	Civilians	29.60	42.90	18.60	2.00	6.90	56.10	28.90	15.00	68.80	49.70	50.30	34.02	89,357.77
Hillsborough County Sheriff, FL	Officers	70.43*	16.00*	8.86*	3.61	1.10*	11.84*	38.75*	49.41*	60.00*	82.90*	17.10*	38.00	98,909.08*
	Civilians	49.60	29.60	13.80	3.00	3.90	37.30	32.10	30.60	76.40	48.90	51.10	38.66	82,399.32
Honolulu PD, HI	Officers	11.39*	1.49*	1.34*	32.00*	53.79	22.44*	14.02*	63.55*	68.60*	86.83*	13.17*	52.00*	123,780.11*
	Civilians	15.40	7.30	2.00	23.00	52.30	38.90	19.00	42.10	72.80	49.80	50.20	42.36	102,709.63
Houston PD, TX	Officers	41.07*	30.19*	20.52	0.39*	7.84	41.25*	39.15*	19.59*	71.14*	82.76*	17.24*	47.00*	111,168.35*
	Civilians	27.80	41.00	21.10	1.90	8.20	58.10	27.00	14.80	69.30	49.60	50.40	33.85	88,784.95
Indianapolis PD, IN	Officers	82.32*	2.44*	6.93*	8.01*	0.29*	15.71*	42.80*	41.49	67.18*	85.68*	14.32*	50.00*	105,491.72*
	Civilians	54.90	10.30	28.10	3.40	3.40	43.20	17.70	39.10	62.60	48.20	51.80	34.94	69,007.27

Agency		White (%)	Hispanic (%)	Black (%)	Other/ unknown race (%)	Asian (%)	Democratic (%)	Republican (%)	Other/ unknown party (%)	Turnout (voting age population)	Male (%)	Female (%)	Age (years)	Household income ($)
Jacksonville County Sheriff, FL	Officers	73.02*	6.97*	15.87*	1.02*	3.12*	15.32*	51.08*	33.59*	70.37*	83.95*	16.05*	39.00*	98,909.54*
	Civilians	51.70	9.90	30.10	3.70	4.70	42.30	34.60	23.20	73.60	48.40	51.60	36.62	75,331.11
Jefferson Parish Sheriff, LA	Officers	52.21	5.68*	32.13*	9.11*	0.87*	30.74*	32.46	36.81*	65.02*	74.37*	25.63*	46.00*	86,885.06*
	Civilians	53.60	12.60	27.00	2.40	4.40	39.30	31.20	29.40	71.10	48.30	51.70	40.38	75,496.01
Jersey City PD, NJ	Officers	42.25*	38.37*	12.92*	0.20*	6.26*	40.56*	13.62*	45.83*	56.56*	83.60*	16.40*	39.00*	106,534.42*
	Civilians	21.90	28.50	21.10	3.60	24.90	56.20	7.80	36.00	64.90	49.60	50.40	34.76	99,941.83
Kansas City PD, MO	Officers	76.93*	5.44*	11.59*	5.33*	0.71*	23.07*	49.40*	27.53*	78.56*	85.64*	14.36*	44.00*	109,277.94*
	Civilians	57.30	10.10	26.30	3.70	2.60	46.80	33.80	19.40	71.20	48.60	51.40	36.14	79,082.43
King County Sheriff, WA	Officers	74.57*	6.26*	5.99	7.46	5.73*	35.69*	32.76*	31.56*	83.09	88.15*	11.85*	44.00*	133,286.34
	Civilians	61.80	8.70	5.70	6.60	17.20	58.00	19.50	22.40	85.20	49.70	50.30	39.91	133,919.76
Las Vegas Metro PD, NV	Officers	59.14*	20.98*	6.61*	8.13*	5.14*	14.97*	47.10*	37.93*	73.30*	85.43*	14.57*	37.00*	114,793.54*
	Civilians	44.20	32.10	11.50	5.30	6.90	40.30	28.20	31.50	69.70	50.00	50.00	38.57	82,764.96
Long Beach PD, CA	Officers	46.03*	38.65*	5.56*	0.27*	9.48*	27.26*	38.83*	33.91*	75.48	87.97*	12.03*	42.00*	123,735.21*
	Civilians	28.20	42.60	12.20	4.20	12.80	52.90	16.90	30.10	73.90	49.40	50.60	35.61	83,535.95
Los Angeles County Sheriff, CA	Officers	28.82*	55.27*	7.06*	3.15*	5.70*	27.29*	38.31*	34.40*	73.77*	81.53*	18.47*	47.00*	121,402.38*
	Civilians	21.20	52.40	8.20	2.70	15.50	49.40	20.70	29.90	75.50	49.40	50.60	37.18	94,900.95
Los Angeles PD, CA	Officers	29.50*	49.93*	9.43*	0.70*	10.44*	34.34*	32.15*	33.50*	75.26*	81.39*	18.61*	45.00*	113,559.23*
	Civilians	28.60	48.30	8.60	3.00	11.50	57.10	12.90	30.00	73.40	49.50	50.50	36.16	91,558.33
Louisville Metro PD, KY	Officers	82.42*	2.51*	12.99*	0.48*	1.60*	30.06*	49.81*	20.14*	8.27*	86.01*	13.99*	44.00*	101,029.21*
	Civilians	59.00	4.70	30.30	3.10	2.90	68.10	22.20	9.70	NaN	48.30	51.70	37.07	63,315.77
Maricopa County Sheriff, AZ	Officers	72.79*	21.22*	3.65	0.52*	1.82	15.62*	52.34*	32.03	79.69*	94.92*	5.08*	49.00*	105,564.36*
	Civilians	77.60	12.60	2.50	4.90	2.40	22.30	47.90	29.90	86.00	47.70	52.30	51.25	98,345.95
Memphis PD, TN	Officers	40.96*	2.40*	55.84*	0.00*	0.80*	32.35*	24.16*	43.48*	71.54*	82.28*	17.72*	48.00*	95,219.09*
	Civilians	27.10	7.00	62.40	1.70	1.90	37.00	11.90	51.00	63.10	47.10	52.90	34.91	63,789.12
Mesa PD, AZ	Officers	77.17*	15.37*	3.29	1.43*	2.74	9.33*	54.23*	36.44	70.47*	87.38*	12.62*	45.00*	113,803.71*
	Civilians	62.40	27.00	3.80	4.70	2.10	26.60	39.50	33.90	78.50	49.40	50.60	38.57	79,346.04
Metro Nashville PD And Sheriff, TN	Officers	81.84*	2.07*	11.01*	3.86	1.21*	14.12*	31.82*	54.06*	69.45*	89.05*	10.95*	42.00*	108,840.14*
	Civilians	56.10	10.30	26.90	3.00	3.60	35.40	17.20	47.40	73.70	48.10	51.90	35.33	85,892.17
Miami PD, FL	Officers	7.37*	66.67*	24.93*	0.45	0.60	28.79*	30.58*	40.62*	68.53*	78.72*	21.28*	39.00*	94,036.36*
	Civilians	10.80	70.70	16.90	0.70	0.90	45.50	22.60	31.90	71.20	49.40	50.60	40.24	62,758.97
Miami-Dade PD, FL	Officers	15.28*	60.40*	22.92*	0.00*	1.40*	24.11*	29.77	46.12*	60.57*	75.00*	25.00*	47.00*	99,395.11*
	Civilians	11.60	70.50	15.20	1.00	1.80	38.00	29.80	32.20	75.70	48.40	51.60	39.89	79,002.34
Milwaukee PD, WI	Officers	65.61*	13.81*	17.28*	1.25*	2.05*	14.84*	10.57*	74.59*	21.15*	83.91*	16.09*	50.00*	88,312.78*
	Civilians	35.80	18.80	37.80	3.40	4.20	63.40	8.80	27.80	42.50	48.10	51.90	32.43	56,810.28
Minneapolis PD, MN	Officers	73.05*	6.16*	9.13*	4.73	6.93	20.68*	33.88*	45.43*	79.43*	84.27*	15.73*	43.00*	123,532.79*
	Civilians	60.00	9.60	18.90	5.60	5.90	83.30	6.70	10.00	88.80	50.60	49.40	33.15	86,513.22
Montgomery County PD, MD	Officers	74.06*	8.62*	12.38*	0.16*	4.78*	24.61*	41.69*	33.70*	73.35*	80.64*	19.36*	42.00*	151,632.88*
	Civilians	44.30	19.20	18.40	3.90	14.20	60.70	15.80	23.40	76.60	48.30	51.70	40.57	155,878.84
Nassau County PD, NY	Officers	75.69*	7.71*	4.99*	10.65*	0.96*	17.66*	51.38*	30.95	83.34*	89.28*	10.72*	44.00*	149,027.32*
	Civilians	62.10	14.80	9.90	2.30	11.00	38.70	31.10	30.20	71.50	48.90	51.10	42.21	155,602.15
New Orleans PD, LA	Officers	37.04*	3.12*	52.20*	6.23*	1.42*	40.58*	19.90*	39.52*	65.01*	76.70*	23.30*	43.00*	82,648.64*
	Civilians	30.80	5.50	58.70	2.10	2.90	64.40	10.10	25.50	70.20	47.20	52.80	37.46	71,994.31
New York City PD, NY	Officers	46.26*	29.31	15.26*	0.78*	8.39*	34.73*	23.84*	41.43*	58.44	80.70*	19.30*	39.00*	116,001.53*
	Civilians	32.10	29.10	21.80	3.00	14.00	67.40	10.10	22.50	58.60	47.60	52.40	37.35	97,203.36
Newark PD, NJ	Officers	13.48*	36.60	27.64*	21.78*	0.50*	39.36*	11.89*	48.74*	55.36*	76.05*	23.95*	42.00*	97,160.86*
	Civilians	10.90	36.50	48.10	2.70	1.80	55.90	4.20	39.90	49.70	48.30	51.70	34.47	52,205.17
Norfolk PD, VA	Officers	69.22*	7.18	18.82*	0.64*	4.15	31.42*	26.16*	42.42*	63.96*	88.68*	11.32*	40.00*	100,074.30*
	Civilians	42.40	7.20	42.30	4.50	3.50	64.40	15.00	20.60	69.30	50.30	49.70	33.85	70,929.58
Oakland County Sheriff, MI	Officers	86.04*	4.17*	8.90*	0.23*	0.68*	38.85*	33.22*	27.93*	76.69*	85.47*	14.53*	44.00*	105,111.82*
	Civilians	57.40	10.00	22.20	3.30	7.10	65.40	23.00	11.60	69.80	48.20	51.80	38.55	92,462.99
Oakland PD, CA	Officers	33.99*	28.21	16.67*	3.07*	18.06*	30.45*	19.18*	50.37*	58.94*	84.92*	15.08*	40.00*	142,625.87*
	Civilians	28.30	27.00	23.20	6.10	15.30	70.20	4.10	25.80	79.70	48.30	51.70	36.78	104,486.40
Oklahoma City PD, OK	Officers	81.48*	6.82*	6.50*	4.17*	1.04*	13.39*	65.44*	21.17	71.77	88.61*	11.39*	43.00*	107,762.39*
	Civilians	56.40	18.20	12.90	8.40	4.20	35.40	45.40	19.20	72.30	49.20	50.80	35.20	80,933.52
Omaha PD, NE	Officers	80.13*	6.33*	2.41*	10.38*	0.76*	12.90*	52.24*	34.86*	76.61	84.59*	15.41*	42.00*	116,319.84*
	Civilians	68.60	12.80	11.30	3.40	3.90	39.20	35.20	25.60	78.30	49.40	50.60	35.08	89,033.82
Orange County Sheriff, CA	Officers	55.70*	29.12*	3.29*	2.98*	8.90*	20.21*	48.44*	31.34*	79.07*	86.73*	13.27*	43.00	129,489.68*
	Civilians	58.00	20.80	1.30	4.20	15.70	31.50	40.00	28.50	89.30	48.70	51.30	42.92	146,606.01
Orange County Sheriff, FL	Officers	60.34*	21.85*	13.61*	2.41*	1.79*	15.09*	34.91*	50.00*	57.85*	84.45*	15.55*	36.00	93,363.26*
	Civilians	38.50	32.00	20.00	3.70	5.80	42.40	25.00	32.60	73.50	49.20	50.80	35.45	84,691.67
Orlando PD, FL	Officers	58.38*	23.62*	14.75*	0.75*	2.50*	17.50*	32.88*	49.62*	58.75*	84.75*	15.25*	39.00*	97,103.17*
	Civilians	36.40	33.20	23.40	3.00	4.00	47.40	21.20	31.40	71.60	48.50	51.50	35.26	73,921.74
Palm Beach County Sheriff, FL	Officers	67.39*	18.23*	12.61*	0.49*	1.28*	18.33*	37.54*	44.14*	67.09*	86.40*	13.60*	42.00*	111,594.87*
	Civilians	51.40	24.10	19.40	2.30	2.80	43.70	26.40	29.90	77.50	48.40	51.60	44.59	91,846.96
Philadelphia PD, PA	Officers	56.97*	9.86*	30.70*	0.48*	2.00*	47.54*	33.37*	19.09*	78.52*	78.42*	21.58*	46.00*	101,931.92*
	Civilians	34.50	14.70	40.80	2.80	7.20	76.40	11.50	12.10	72.80	47.30	52.70	35.39	65,363.44
Phoenix PD, AZ	Officers	71.11*	19.69*	3.94*	2.68*	2.59*	17.38*	44.38*	38.24*	74.17*	85.87*	14.13*	48.00*	107,438.95*
	Civilians	42.80	42.50	6.60	4.60	3.60	38.00	27.50	34.40	75.70	49.80	50.20	34.30	78,537.91
Pinellas County Sheriff, FL	Officers	77.74*	6.89	13.84*	0.25*	1.29*	15.93*	43.85*	40.22*	70.05*	84.69*	15.31*	44.00*	92,627.00*
	Civilians	81.40	7.70	4.30	3.10	3.50	30.50	39.90	29.60	81.40	48.00	52.00	49.91	84,030.27
Pittsburgh PD, PA	Officers	84.98*	1.26*	11.87*	1.16*	0.74*	40.55*	44.22*	15.23	86.13*	85.82*	14.18*	39.00*	98,137.54*
	Civilians	64.70	3.20	22.70	3.60	5.80	71.60	13.50	14.90	72.70	48.70	51.30	34.73	72,381.50
Portland Police Bureau, OR	Officers	82.02*	5.22*	3.83*	2.90*	6.03*	24.13*	27.73*	48.14*	70.65*	82.71*	17.29*	43.00*	120,769.68*
	Civilians	70.50	10.10	5.40	5.90	8.10	53.40	9.60	37.10	73.80	49.50	50.50	37.93	97,193.34
Prince Georges County PD, MD	Officers	41.97*	10.57*	43.34*	0.20*	3.92	44.13*	25.52*	30.35*	66.25*	85.38*	14.62*	40.00*	138,893.52*

Agency		White (%)	Hispanic (%)	Black (%)	Other/ unknown race (%)	Asian (%)	Democratic (%)	Republican (%)	Other/ unknown party (%)	Turnout (voting age population)	Male (%)	Female (%)	Age (years)	Household income ($)
	Civilians	12.70	18.40	61.70	3.10	4.10	78.50	6.40	15.10	71.00	48.10	51.90	38.14	102,998.63
Raleigh PD, NC	Officers	81.67*	5.56*	9.86*	1.25*	1.67*	15.83*	39.72*	44.44*	85.14	89.17*	10.83*	40.00*	107,125.53*
	Civilians	55.20	11.00	26.40	2.90	4.50	42.50	19.80	37.70	84.00	48.40	51.60	35.80	96,560.05
Richmond PD, VA	Officers	59.09*	5.00*	27.05*	7.05*	1.82	43.76*	29.17*	27.06*	72.17	82.53*	17.47*	48.00*	109,578.36*
	Civilians	40.90	7.00	46.50	3.60	2.00	74.60	9.60	15.80	70.70	47.70	52.30	35.70	73,864.18
Riverside County Sheriff, CA	Officers	50.65*	34.57*	3.89*	6.96*	3.94*	22.85*	43.21*	33.94*	77.39*	89.49*	10.51*	43.00*	114,417.27*
	Civilians	35.40	48.80	6.10	3.30	6.30	39.70	32.20	28.00	79.60	49.70	50.30	36.60	89,235.25
Rochester PD, NY	Officers	74.55*	11.74*	10.91*	0.66*	2.15	14.71*	55.04*	30.25*	77.69*	85.45*	14.55*	40.00*	102,547.01*
	Civilians	37.90	18.90	36.90	3.40	2.90	64.20	9.90	26.00	46.60	48.50	51.50	33.24	51,660.92
Sacramento County Sheriff, CA	Officers	66.13*	16.59*	4.75*	2.63*	9.90*	21.80*	46.68*	31.52*	82.04*	83.70*	16.30*	45.00*	124,007.91*
	Civilians	50.60	21.30	8.40	7.30	12.50	41.30	29.90	28.80	84.00	48.50	51.50	37.14	84,117.66
Sacramento PD, CA	Officers	69.12*	13.08*	5.35*	3.11*	9.34*	16.31*	49.69*	34.00*	81.20	83.19*	16.81*	43.00*	136,215.84*
	Civilians	31.80	29.30	12.80	7.50	18.60	55.40	15.50	29.20	82.60	48.90	51.10	35.41	80,100.12
St. Louis Metro PD, MO	Officers	65.96*	2.03*	30.48*	0.59*	0.93*	40.05*	39.97*	19.98*	75.53*	83.49*	16.51*	44.00*	101,782.60*
	Civilians	43.60	4.00	46.20	2.80	3.30	85.20	11.30	3.50	67.50	48.40	51.60	36.74	62,162.18
San Antonio PD, TX	Officers	37.69*	54.98*	4.70*	1.53	1.10*	40.81*	41.23*	17.96*	73.85*	88.52*	11.48*	48.00*	101,306.88*
	Civilians	26.70	61.70	6.70	2.10	2.70	62.90	24.20	12.90	67.80	49.40	50.60	34.32	75,298.32
San Bernardino County Sheriff, CA	Officers	53.26*	34.12*	5.06*	4.66*	2.90*	27.37*	42.37*	30.26	74.87*	85.25*	14.75*	43.00*	107,251.53*
	Civilians	37.70	42.90	7.30	3.70	8.40	36.30	34.30	29.40	77.20	49.80	50.20	35.31	83,483.57
San Diego County Sheriff, CA	Officers	53.25	32.49	5.48*	0.66*	8.12*	19.68*	47.17*	33.15*	79.54*	81.23*	18.77*	41.00*	127,554.58*
	Civilians	55.00	30.40	3.70	4.40	6.40	33.80	35.60	30.60	84.70	50.60	49.40	38.48	109,814.86
San Diego PD, CA	Officers	59.39*	25.45*	5.74	4.87	4.55*	20.90*	46.40*	32.70	84.08	83.70*	16.30*	44.00*	130,034.69*
	Civilians	42.80	29.90	6.10	4.50	16.80	45.40	21.30	33.30	82.90	50.40	49.60	36.27	108,601.61
San Francisco PD, CA	Officers	47.55*	17.72*	9.58*	1.92*	23.24*	27.82*	17.62*	54.57*	59.22*	85.49*	14.51*	43.00*	156,316.63
	Civilians	40.50	15.20	5.00	5.20	34.10	62.50	6.80	30.80	86.50	51.00	49.00	39.29	157,990.14
San Jose PD, CA	Officers	46.33*	28.47*	1.95*	10.53*	12.71*	32.63*	27.93*	39.44*	74.25*	86.92*	13.08*	43.00*	156,770.67*
	Civilians	27.10	31.20	2.80	4.20	34.80	50.00	17.10	32.90	83.40	50.50	49.50	37.59	142,187.18
Seattle PD, WA	Officers	67.80*	5.47*	7.98	10.78*	7.98*	29.76*	36.26*	33.97*	78.58*	84.79*	15.21*	50.00*	142,190.79*
	Civilians	63.70	6.80	7.20	7.00	15.30	75.20	5.50	19.30	86.20	50.60	49.40	36.47	128,545.84
St Louis County PD, MO	Officers	86.00*	1.92	10.38*	0.11*	1.58*	31.49*	45.82*	22.69*	77.77	83.97*	16.03*	41.00*	105,515.56*
	Civilians	70.70	2.00	22.30	2.40	2.70	56.40	38.00	5.60	75.80	47.70	52.30	42.09	92,985.21
Suffolk County PD, NY	Officers	84.94*	10.18*	2.67*	1.15*	1.07*	15.76*	46.41*	37.83*	85.68*	88.80*	11.20*	47.00*	150,138.86*
	Civilians	67.60	19.30	7.20	2.00	3.90	34.50	30.80	34.70	74.20	49.20	50.80	41.76	129,328.37
Tampa PD, FL	Officers	68.52*	16.90*	12.50*	0.23*	1.85*	13.66*	42.13*	44.21*	67.01*	82.52*	17.48*	42.00*	106,850.47*
	Civilians	43.70	27.20	22.10	2.90	4.20	46.10	25.30	28.70	74.00	48.80	51.20	36.34	84,284.38
Toledo PD, OH	Officers	76.96*	5.54*	4.29*	12.68*	0.54*	23.55*	31.59*	44.86*	69.45*	83.31*	16.69*	46.00*	90,639.24*
	Civilians	60.10	8.50	25.80	4.30	1.30	46.20	13.80	40.00	65.70	48.20	51.80	36.23	53,321.56
Tucson PD, AZ	Officers	62.07*	31.60*	2.35*	1.43*	2.56	14.11*	46.83*	39.06*	71.68*	84.87*	15.13*	44.00*	99,503.07*
	Civilians	45.40	42.90	4.30	4.40	3.00	44.00	23.50	32.50	74.80	49.20	50.80	35.86	61,498.06
Tulsa PD, OK	Officers	86.75*	3.56*	3.13*	5.56*	1.00*	11.27*	60.28*	28.46*	68.18*	85.48*	14.52*	43.00*	106,118.80*
	Civilians	54.90	16.00	14.50	11.30	3.40	38.80	42.40	18.80	73.60	48.60	51.40	36.17	74,644.31
Ventura County Sheriff, CA	Officers	64.16*	27.93	2.19*	0.38*	5.34*	31.94*	37.08*	30.98	79.50*	85.80*	14.20*	44.00*	121,719.11*
	Civilians	59.90	26.90	1.30	3.60	8.30	39.00	32.60	28.40	88.10	48.80	51.20	42.84	134,713.45
Virginia Beach PD, VA	Officers	82.43*	4.89*	7.27*	2.91*	2.51*	22.32*	42.67*	35.01*	75.17	82.83*	17.17*	40.00*	112,010.98*
	Civilians	61.70	8.10	18.40	5.10	6.60	45.50	33.70	20.80	73.30	49.00	51.00	37.74	97,309.03
Washington DC PD, DC	Officers	34.77*	10.14	50.75*	0.07*	4.27	49.25*	6.93*	43.81*	52.40*	77.00*	23.00*	47.00*	124,423.49
	Civilians	36.60	11.00	45.40	3.10	3.90	77.10	5.50	17.40	69.80	47.40	52.60	34.53	125,850.25
Wayne County Sheriff, MI	Officers	53.85*	4.17	31.41*	9.62*	0.96*	65.17*	14.41*	20.42	66.37*	76.58*	23.42*	42.00	78,755.56*
	Civilians	69.60	3.40	14.80	3.00	9.30	54.50	22.10	23.30	78.20	49.10	50.90	41.98	106,198.82
Wichita PD, KS	Officers	52.77*	5.26*	4.43*	35.87*	1.66*	9.14*	47.37*	43.49*	61.08*	88.50*	11.50*	44.00*	94,709.58*
	Civilians	64.20	16.50	10.20	4.40	4.80	28.40	38.50	33.10	72.30	49.30	50.70	35.88	74,713.90
Yonkers PD, NY	Officers	77.54*	15.02*	6.79*	0.00*	0.65*	20.32*	28.71*	50.97*	60.97*	84.84*	15.16*	43.00*	132,011.10*
	Civilians	36.70	38.30	16.10	2.60	6.30	54.80	17.30	27.90	66.20	47.90	52.10	38.96	90,688.29

Table F.1: **Comparison of Officer and Civilian Traits for all Included Agencies**. The table displays the share of officers and civilians in each jurisdiction with a given attribute. Stars denote a statistically significant difference between officers and civilians.

G Officers' Place of Residence

Even if police do not themselves reflect the communities they serve, they may live in representative neighborhoods, which could facilitate awareness of and empathy for the issues experienced by civilians they encounter on the job (Pettigrew, 1998). In addition, recent work theorizes that the groups with whom officers socialize with off the clock can distort beliefs about other groups' behavior, leading to discriminatory

policing (Little and & Hübert, 2022). Often invoking similar logic, 26 of the 100 largest agencies have adopted policies that encourage or require officers to reside inside their jurisdictions, according to our close examination of police union contracts, hiring webpages, and personnel policies for each jurisdiction. It is clear that numerous top agencies regard officer residency as an important consideration.[24]

To characterize officers' home neighborhoods, we matched officer home addresses from L2—redacted from our replication data for security and privacy reasons—to U.S. Census tracts. We compared the traits of these tracts to the overall jurisdiction. The results are displayed in Table G.1.[25] Officers' home tracts tend to have higher shares of Republicans (+9 p.p.) and White residents (+13 p.p.). They also tend to have a higher median household annual income (+$12,558) and participate in elections at greater rates (+10 p.p. among voting-age population). In the same vein, officers tend to live in areas with lower shares of Black (−7 p.p.) and Hispanic (−5 p.p.) residents than the jurisdiction-wide average.

Table G.2 displays the share of police officers/sheriff's deputies with various attributes, relative to the hypothetical compositions their agencies would have if randomly drawn from their jurisdictions. The table also displays difference in differences testing whether sheriff's deputies are closer on each attribute to their local populations than are police officers. The table indicates that on various key attributes, sheriff's agencies are more similar to their local populations than are police agencies. For example, both types of agencies show overrepresentation of white officers, but the degree of overrepresentation is 5 percentage points larger among police agencies. Likewise, Democrats are underrepresented in both types of agencies, but the underrepresentation is 6 percentage points larger for police agencies. These patterns are consistent with the idea that elections promote descriptive representation in policing, though as police and sheriff's agencies and jurisdictions differ in multiple unobserved ways, a more thorough examination of this causal account would be necessary before drawing that conclusion.

[24]Our complete data for residency rules for each agency can be found here: https://dl.dropboxusercontent.com/s/2se7l3be55bnank/residency_data_table.pdf?dl=0.

[25]This analysis is restricted to the 86% of officers matched to the L2 database, which contains officer addresses.

Table G.1: **Average Attributes of Officers' Home Census Tracts Relative to their Jurisdictions.** The table displays the average characteristics of the U.S. Census Tracts in which police officers reside, the average characteristics of their jurisdictions, and the difference between the two. Census data does not provide means or full distributions for age; we therefore report, median of ages in officers' tracts, median ages in officers' jurisdictions, and the difference in means between the two. **denotes $p<.01$; * denotes $p<.05$; brackets contain 95% confidence intervals. N indicates the number of officers.

Variable	Value	Actual officer (%)	Hypothetical representative officer (%)	Difference	N
Race	White	50.66	38.03	12.62** [12.50, 12.74]	187,952
	Hispanic	23.23	28.11	−4.88** [−4.97, −4.80]	187,952
	Black	14.27	21.16	−6.89** [−6.99, −6.80]	187,952
	Other/unknown race	3.40	3.40	0.01 [−0.00, 0.02]	187,952
	Asian	8.44	9.30	−0.86** [−0.90, −0.81]	187,952
Party (voting age population)	Republican	23.54	14.17	9.36** [9.29, ,9.43]	187,943
	Democratic	39.04	43.27	−4.23** [−4.30, −4.16]	187,943
	Other/unknown party	39.51	42.65	−3.14** [−3.20, −3.08]	187,943
Turnout (voting age population)	General election, 2020	64.25	54.61	9.63** [9.56, 9.71]	185,757
Gender	Male	48.82	48.69	0.12** [0.11, 0.14]	187,952
	Female	51.18	51.31	−0.12** [−0.14, −0.11]	187,952
Age (years)	–	38.80	36.85	2.32** [2.29, 2.35]	187,949
Household income ($)	–	104783.35	92225.06	12558.29** [12363.44, 12753.13]	187,924

Table G.2: **Comparison of Average Officer and Civilian Traits for municipal police agencies ('Officer') and for Sheriff's Offices ('Sheriff').** The table displays, from left to right, the actual share of municipal officers with a given attribute; the share of officers who would have the attribute if taken as a random draw from their jurisdictions; the actual share of Sheriff officers with a given attribute; the share of Sheriff officers who would have the attribute if taken as a random draw from their jurisdictions. Census data does not provide means or full distributions for age; we therefore report, for both police and sheriff's agencies, median of actual officer ages, median age for a hypothetical set of officers with ages equal to the median age in their jurisdiction, and the difference in means between the two. The 'Difference in Difference' column shows the difference between the Officer-Civilian difference and the Sheriff-Civilian difference. **denotes $p<.01$; * denotes $p<.05$; brackets contain 95% confidence intervals. N indicates the number of officers.

Variable	Value	Actual officer (%)	Hypothetical representative officer (%)	Actual Sheriff %	Hypothetical representative sheriff (%)	Difference in difference
Race	White	51.83	37.47	49.00	39.85	5.20** [4.70, 5.70]
	Hispanic	21.83	26.05	31.38	35.68	0.08 [−0.38, 0.53]
	Black	17.28	23.89	11.13	10.76	−6.98** [−7.32, −6.64]
	Other/unknown race	3.56	3.43	3.99	3.36	−0.50** [−0.70, −0.29]
	Asian	5.50	9.16	4.50	10.35	2.18** [1.96, 2.41]
Party (voting age population)	Republican	30.83	12.32	38.92	21.24	0.82** [0.32, 1.33]
	Democratic	32.47	45.95	26.72	33.71	−6.49** [−6.96, −6.02]
	Other/unknown party	36.70	42.03	34.36	45.08	5.39** [4.88, 5.90]
Turnout (voting age population)	General election, 2020	68.49	53.36	72.91	59.62	1.84** [1.37, 2.32]
Gender	Male	82.60	48.58	83.37	49.11	−0.24 [−0.63, 0.15]
	Female	17.40	51.42	16.63	50.89	0.24 [−0.15, 0.63]
Age (years)	–	43.00	36.46	45.00	38.35	0.97** [0.80, 1.14]
Household income ($)	–	114097.10	90990.40	114602.25	97048.46	5552.92** [4867.16, 6238.68]

Table G.3: **Comparison of Chicago Police Officer and Civilian Traits by district.** The table displays the share of officers and civilians in each police district with a given attribute. Stars denote a statistically significant difference between officers and civilians.

District		White (%)	Hispanic (%)	Black (%)	Other/ unknown race (%)	Democratic (%)	Republican (%)	Other party (%)
Albany Park	Officers	0.68*	0.22*	0.04	0.06*	0.43	0.21*	0.22
Albany Park	Civilians	0.40	0.40	0.03	0.16	0.40	0.04	0.25
Austin	Officers	0.56*	0.22*	0.19*	0.04*	0.48*	0.18*	0.27*
Austin	Civilians	0.03	0.09	0.87	0.01	0.89	0.01	0.07
Calumet	Officers	0.34*	0.10*	0.55*	0.01	0.63*	0.11*	0.14*
Calumet	Civilians	0.02	0.04	0.93	0.02	0.96	0.01	0.06
Central	Officers	0.57*	0.13*	0.28*	0.02*	0.56*	0.15*	0.17*
Central	Civilians	0.53	0.07	0.17	0.23	0.38	0.04	0.27
Chicago Lawn	Officers	0.66*	0.25*	0.07*	0.02	0.50	0.16*	0.23
Chicago Lawn	Civilians	0.17	0.62	0.19	0.02	0.48	0.03	0.21
Deering	Officers	0.65*	0.25*	0.08*	0.03*	0.54*	0.22*	0.17*
Deering	Civilians	0.15	0.54	0.10	0.20	0.36	0.02	0.22
Englewood	Officers	0.42*	0.23*	0.32*	0.03*	0.59*	0.12*	0.22*
Englewood	Civilians	0.01	0.06	0.91	0.01	0.97	0.01	0.07
Grand Central	Officers	0.66*	0.24*	0.05*	0.04	0.46*	0.19*	0.25
Grand Central	Civilians	0.15	0.69	0.13	0.03	0.42	0.03	0.27
Grand Crossing	Officers	0.27*	0.18*	0.53*	0.02	0.63*	0.09*	0.19*
Grand Crossing	Civilians	0.04	0.03	0.90	0.03	0.84	0.01	0.05
Gresham	Officers	0.30*	0.19*	0.49*	0.02	0.61*	0.10*	0.21*
Gresham	Civilians	0.01	0.02	0.95	0.02	0.94	0.01	0.04
Harrison	Officers	0.53*	0.25*	0.18*	0.04*	0.49*	0.14*	0.29*
Harrison	Civilians	0.04	0.16	0.77	0.02	0.84	0.01	0.13
Jefferson Park	Officers	0.81*	0.14*	0.03*	0.03*	0.44	0.24*	0.17*
Jefferson Park	Civilians	0.63	0.27	0.01	0.09	0.41	0.09	0.29
Lincoln	Officers	0.70*	0.15	0.06*	0.09*	0.47	0.18*	0.21
Lincoln	Civilians	0.55	0.18	0.09	0.18	0.48	0.04	0.24
Morgan Park	Officers	0.60*	0.11*	0.28*	0.01*	0.59*	0.16*	0.15*
Morgan Park	Civilians	0.34	0.05	0.58	0.03	0.86	0.05	0.10
Near North	Officers	0.61*	0.15*	0.19*	0.04*	0.52*	0.15*	0.22*
Near North	Civilians	0.73	0.06	0.07	0.15	0.35	0.08	0.34
Near West	Officers	0.53*	0.33*	0.11*	0.02*	0.53*	0.16*	0.24*
Near West	Civilians	0.46	0.26	0.17	0.12	0.47	0.04	0.32
Ogden	Officers	0.41*	0.51*	0.07*	0.02	0.48	0.17*	0.27*
Ogden	Civilians	0.05	0.64	0.30	0.01	0.47	0.01	0.18
Rogers Park	Officers	0.73*	0.15*	0.05*	0.07*	0.48*	0.21*	0.19*
Rogers Park	Civilians	0.44	0.19	0.18	0.19	0.42	0.03	0.25
Shakespeare	Officers	0.51	0.37	0.06	0.06	0.44	0.15*	0.28*
Shakespeare	Civilians	0.53	0.35	0.05	0.07	0.46	0.04	0.33
South Chicago	Officers	0.48*	0.22*	0.29*	0.02	0.55*	0.14*	0.21*
South Chicago	Civilians	0.07	0.30	0.62	0.01	0.73	0.02	0.14
Town Hall	Officers	0.62*	0.23*	0.09*	0.06*	0.47	0.18*	0.23*
Town Hall	Civilians	0.74	0.10	0.06	0.10	0.45	0.05	0.31
Wentworth	Officers	0.22	0.14*	0.62*	0.02*	0.68*	0.08*	0.16*
Wentworth	Civilians	0.19	0.04	0.66	0.11	0.73	0.01	0.11

Table G.4: **Comparison of Houston Police Officer and Civilian Traits by division.** The table displays the share of officers and civilians in each police district with a given attribute. Stars denote a statistically significant difference between officers and civilians. Two police districts where the jurisdiction was an airport ('Airport-Hobby Division' and 'Airport-IAH Division') were excluded due to a lack of a civilian comparison.

Division		White (%)	Hispanic (%)	Black (%)	Other/ unknown race (%)	Democratic (%)	Republican (%)	Other party (%)
Central Division	Officers	0.34*	0.38*	0.09	0.14	0.46	0.31*	0.15
Central Division	Civilians	0.57	0.27	0.06	0.10	0.41	0.22	0.17
Clear Lake Division	Officers	0.43*	0.34*	0.04*	0.16	0.38	0.35*	0.19*
Clear Lake Division	Civilians	0.28	0.49	0.12	0.12	0.33	0.19	0.11
Eastside Division	Officers	0.27*	0.49*	0.07*	0.10*	0.49	0.23*	0.22*
Eastside Division	Civilians	0.06	0.90	0.03	0.02	0.42	0.04	0.06
Kingwood Division	Officers	0.42*	0.36*	0.11	0.11	0.33	0.50	0.14
Kingwood Division	Civilians	0.68	0.19	0.07	0.06	0.30	0.50	0.12
Midwest Division	Officers	0.35	0.24*	0.07*	0.12	0.43*	0.30*	0.22*
Midwest Division	Civilians	0.37	0.36	0.12	0.15	0.25	0.18	0.12
North Belt Division	Officers	0.48*	0.24*	0.05*	0.24*	0.38	0.24*	0.19
North Belt Division	Civilians	0.08	0.57	0.30	0.05	0.33	0.03	0.04
North Division	Officers	0.50*	0.30*	0.06*	0.12*	0.42	0.36*	0.16*
North Division	Civilians	0.16	0.62	0.20	0.03	0.48	0.10	0.06
Northeast Division	Officers	0.49*	0.26*	0.08*	0.14*	0.39*	0.37*	0.17*
Northeast Division	Civilians	0.05	0.56	0.38	0.01	0.59	0.02	0.04
Northwest Division	Officers	0.39*	0.30*	0.05*	0.13*	0.39*	0.45*	0.09
Northwest Division	Civilians	0.28	0.56	0.09	0.07	0.26	0.16	0.09
South Central Division	Officers	0.55*	0.19*	0.12*	0.11	0.40*	0.39*	0.16
South Central Division	Civilians	0.33	0.28	0.28	0.11	0.49	0.10	0.12
South Gessner Division	Officers	0.38*	0.27*	0.14*	0.16*	0.47*	0.34*	0.12*
South Gessner Division	Civilians	0.12	0.55	0.26	0.08	0.32	0.06	0.05
Southeast Division	Officers	0.41*	0.34*	0.13*	0.10*	0.49*	0.25*	0.18*
Southeast Division	Civilians	0.04	0.47	0.45	0.03	0.61	0.03	0.04
Southwest Division	Officers	0.34	0.29	0.16*	0.09	0.50	0.29*	0.14
Southwest Division	Civilians	0.26	0.34	0.28	0.11	0.59	0.13	0.09
Westside Division	Officers	0.31	0.27*	0.06*	0.14	0.35*	0.35*	0.21*
Westside Division	Civilians	0.28	0.35	0.21	0.16	0.28	0.16	0.10

H Measurement Error in Race/Ethnicity

Imputed L2 race and ethnicity variables are used for 14 percent of agencies, which contain approximately 8% of our officers. To get a sense of the scale of the potential for mismeasurement in the L2 race data, we compare the shares of each racial/ethnic group as measured in LEMAS vs. L2 for the agencies found in both data sets.

The table below, Table H.1, displays the proportion of officers in each racial/ethnic category as measured by L2 vs. LEMAS. As the table shows, among these agencies, L2 underrepresents the share of officers who are white by 13 percentage points, on average. L2 also under-represents racial and ethnic minorities relative to LEMAS. The main discrepancy stems from the "other/unknown" category, which is 22% in L2 but only 2% in LEMAS (2020).

The following table, Table H.2 shows the comparison between officers and civilians after adjusting for the measurement error shown in Table H.1 for agencies that are not covered by the LEMAS data. Because 92% of our officers being in agencies covered by LEMAS, results are nearly identical to Table 1.

I Measurement Error in Party ID

At a high level, there are two potential sources of measurement error in our method for ascertaining officers' party identification: (i) officers who have partisan identities are erroneously not matched to the voter file, and (ii) officers are matched to the voter file but their party identification is mismeasured, which could occur due to matching to the wrong individual, erroneous imputation, or "stale" registrations. To address these issues, we engage in a series of bounding exercises assuming conservative assumptions about the nature of measurement error, employ an alternate measure of party identification based on recent primary participation, and subset to states where voters can identify which party they are affiliated with on their voter registration forms.

To address measurement error due to a failure to match officers to L2, we include an extensive best- and worst-case bounding exercise which evaluate the hypothetical impact of all unmatched officers being Democrats or Republicans (see Table I.1 below). Even using the most conservative worst case scenario for the officers who are not matched to the voter file, officers overall are still far more likely to be Republican than civilians in their jurisdictions. This exercise also shows that under this worst-case measurement error scenario, we cannot reject the possibility that Democrats are slightly overrepresented on police forces by 2 percentage points. We note this test is extremely conservative, as it assumes all unmatched officers identify

Table H.1: Comparison of Average Officer Race when using LEMAS Compared to using L2 for the 86% of Agencies (Covering 92% of Officers) with LEMAS data.

Race (%)	Data from L2	Data from LEMAS	Change (%)
White	44.72	50.57	13.08
Hispanic	19.95	25.02	25.41
Black	10.52	16.69	58.69
Other/unknown	21.71	2.12	−90.26
Asian	3.10	5.61	80.75

Table H.2: **Comparison of Average Officer and Civilian Race Variables after Approximate Debiasing of L2 Race Data.** L2 race estimates are used for 8% of officers (14% of agencies). However, as Table H.1 shows, L2 race estimates are in general not well-calibrated. In this analysis, we adjust L2 estimates by taking the proportion of officers of each race, only among agencies with only L2 race data, and shifting it based on estimated misclassification rates in agencies where LEMAS-based ground truth is available. For example, Table H.1 shows that when LEMAS ground-truth race data is available, L2 undercounts the share of White officers by 13%. Here, for agencies where only L2 is available, we therefore inflate the share of White officers by a corresponding factor. Agencies in which LEMAS race data is available are unchanged. The table displays, from left to right, the actual share of officers with a given attribute; the share of officers who would have the attribute if taken as a random draw from their jurisdictions; and the difference between the two. The Difference column is made by taking the difference between the officer and civilian trait. **denotes $p < .01$; * denotes $p < .05$; brackets contain 95% confidence intervals. denotes. N indicates number of officers.

Variable	Value	Actual officer (%)	Hypothetical representative officer (%)	Difference	N
Race	White	52.07	37.95	14.12**	114,216
				[13.92, 14.32]	
	Hispanic	23.82	27.98	−4.16**	52,247
				[−4.33, −3.99]	
	Black	16.52	21.26	−4.75**	36,229
				[−4.90, −4.60]	
	Other/unknown race	2.20	3.42	−1.22**	4,821
				[−1.28, −1.16]	
	Asian	5.40	9.40	−3.99**	11,856
				[−4.08, −3.90]	

with one of the two major parties, when in reality at least some share identify as pure independents or with a minor party. Because of this, we view it as extremely unlikely that the worst-case estimate is correct.

To address measurement error due to mismatching, we first re-compute our core results using an alternate threshold for the posterior probability of a correct match of 0.95 (see Table I.2 below). As the table shows, our core conclusions remain virtually unaffected. Second, we employ an alternate measure of party ID: the most recent party primary a voter participated in, according to L2 (see Table I.3 below). This approach has the simultaneous benefit of using a recent measure of party identification, which partially addresses concerns over "stale" registrations, while avoiding reliance on imputed measures. If officers and civilians did not participate in any primaries on record, we code them as "other/unknown" party for this test. Table I.3 shows our core results using L2's imputed party identification measure, while the bottom table shows results using the most recent primary alternative measure. As the table shows, while this alternate measure changes the base rates of party ID, our overall conclusion that Republicans are substantially overrepresented holds.

As a further check, we also re-compute core results after subsetting to states where voters are allowed to indicate which political party they are affiliated with when registering to vote and where L2 is presumably less reliant on imputation. These results, shown in Table I.4 below, are consistent with our core conclusions in terms of the disparities between officers and civilians.

Next, we consider the potential for mismeasurement in party identification due to erroneous matches in the voter file in the case of multiple high probability matches. To evaluate the potential scale of this problem for our study, we conducted a bounding exercise assuming best/worst case scenarios for officers with multiple matches. Specifically, we re-compute core results assuming that every officer with a multiple match was erroneously paired with an individual of a different party identification. As Table I.5 below shows, these extremely conservative assumptions lead to very wide bounds. For example, under these best/worst case scenarios, the difference in the share Republican among officers and civilians ranges between 9 and 34 percentage points. For Democrats, it ranges from -25 to 2 percentage points. In other words, even under the most extreme scenarios possible, we can definitively conclude that officers are more heavily Republican compared to representative civilians, but we cannot draw firm conclusions about the share of Democratic officers.

However, using an anonymous reviewer's helpful suggestion to incorporate additional information such as age in the merge procedure, we are able to gain a more realistic portrait of the potential severity of measurement error here. In addition to name-only matching, we conduct a validation exercise with 20 agencies where officer age is also available (Table I.6). In addition, we conduct the same exercise using the three agencies which include the officer's exact date of birth (Table I.7). We find that results are nearly identical when using name-only as when using name+age or name+date-of-birth.

Taken together, we believe that i) the substantial reduction in duplicate matches we see when incorporating additional merge information combined with ii) the near-identical results we obtain when doing so, demonstrates that our central conclusions are not being driven by erroneous record linkages.[26]

[26]Incorporating age when matching reduces the number of officers with more than one potential match from 38% of officers to 16%; using date of birth rate than age reduces the multiple-match officers even further to only 2% of officers.

Table I.1: **Average Officer Traits Relative to Jurisdictions (Estimated Bounds Based on Extreme Values for Unmatched Officers).** The table displays, from left to right, the lowest possible share of officers with a given attribute; the largest share of officers with a given attribute; the share of officers who would have the attribute if taken as a random draw from their jurisdictions; and the differences between this hypothetical share and the lower and upper bounds. Lower and upper bounds are computed by assigning maximally extreme values to officers not observable in any of our data sources (e.g. that no unmatched officers are Democrats, or that all are Democrats). "Difference" columns report the gap between the hypothetical representative value and these upper/lower bounds. **denotes $p<.01$; * denotes $p<.05$.

Variable	Value	Officer lower bound (%)	Officer upper bound (%)	Hypothetical representative officer (%)	Difference lower bound	Difference upper bound
Race	White	51.26	52.24	37.95	13.31**	14.29**
	Hispanic	23.75	24.73	27.98	−4.23**	−3.25**
	Black	16.05	17.03	21.26	−5.21**	−4.23**
	Other/unknown race	2.66	3.65	3.42	−0.76**	0.23**
	Asian	5.30	6.28	9.40	−4.10**	−3.11**
Party (Voting age population)	Republican	32.45	46.14	14.11	18.34**	32.03**
	Democratic	31.32	45.01	43.50	−12.18**	1.52**
	Other/unknown party	22.54	36.23	42.64	−20.10**	−6.41**
Turnout (voting age population)	General election, 2020	69.39	83.15	54.62	14.76**	28.52**

Table I.2: **Comparison of Average Officer and Civilian Traits (0.95 Match Probability Threshold).** Note: The table displays, from left to right, the attributes of actual officers (means, except where otherwise noted) with a given attribute; the attributes of a hypothetical set of officers randomly drawn from their respective jurisdictions; and the difference between the two. Census data does not provide means or full distributions for age; we therefore report, median of actual officer ages, median age for a hypothetical set of officers with ages equal to the median age in their jurisdiction, and the difference in means between the two. **denotes $p<.01$; * denotes $p<.05$; brackets contain 95% confidence intervals. N indicates the number of officers.

Variable	Value	Actual officer (%)	Hypothetical representative officer (%)	Difference	N
Race	White	50.78	37.95	12.83** [12.63, 13.03]	111,391
	Hispanic	23.53	27.98	−4.44** [−4.61, −4.28]	51,623
	Black	15.99	21.26	−5.27** [−5.42, −5.13]	35,072
	Other/unknown race	4.49	3.42	1.07** [0.98, 1.15]	9,843
	Asian	5.21	9.40	−4.18** [−4.27, −4.09]	11,438
Party (voting age pop.)	Republican	25.50	14.11	11.39** [11.21, 11.57]	55,928
	Democratic	22.86	43.50	−20.64** [−20.82, −20.47]	50,136
	Other/unknown party	51.65	42.64	9.01** [8.81, 9.21]	113,301
Turnout (voting age population)	General election, 2020	52.04	54.62	−2.58** [−2.79, −2.38]	112,953
Gender	Male	82.75	48.69	34.07** [33.91, 34.22]	181,532
	Female	17.25	51.31	−34.07** [−34.22, −33.91]	37,833
Age (years)	–	44.00	36.94	7.87** [7.80, 7.94]	139,135
Household income ($)	–	115131.11	92002.72	23 128.39** [22812.62, 23444.15]	138,631

Table I.3: **Comparison of Officer and Civilian Party Identification.** Top panel reports L2-estimated party identification; bottom panel reports party based on the most recent primary in which an individual voted. The table displays, from left to right, the actual share of officers with a given attribute; the share of officers who would have the attribute if taken as a random draw from their jurisdictions; and the difference between the two. The Difference column is made by taking the difference between the officer and civilian trait. **denotes $p < .01$; * denotes $p < .05$; brackets contain 95% confidence intervals. denotes. N indicates number of officers.

Variable	Value	Actual officer (%)	Hypothetical representative officer (%)	Difference	N
Party (voting age population)	Republican	32.45	14.11	18.34**	71,177
				[18.15, 18.53]	
	Democratic	31.32	43.50	−12.18**	68,705
				[−12.37, −11.99]	
	Other/unknown party	36.23	42.64	−6.41**	79,483
				[−6.61, −6.20]	

(a) **Party ID as identified by L2**

Variable	Value	Actual officer (%)	Hypothetical representative officer (%)	Difference	N
Party (voting age population)	Republican	20.49	8.10	12.39**	44,940
				[12.22, 12.55]	
	Democratic	22.26	25.21	−2.96**	48,825
				[−3.13, −2.79]	
	Other/unknown party	57.26	66.93	−9.68**	125,600
				[−9.88, −9.47]	

(b) **Party ID based on the most recent party primary election**

Table I.4: **Comparison of Average Officer and Civilian Traits for States with Partisan Affiliations Recorded for Registered Voters.** The table displays, from left to right, the attributes of actual officers (means, except where otherwise noted) with a given attribute; the attributes of a hypothetical set of officers randomly drawn from their respective jurisdictions; and the difference between the two. Census data does not provide means or full distributions for age; we therefore report, median of actual officer ages, median age for a hypothetical set of officers with ages equal to the median age in their jurisdiction, and the difference in means between the two. **denotes $p<.01$; * denotes $p<.05$; brackets contain 95% confidence intervals. N indicates the number of officers.

Variable	Value	Actual officer (%)	Hypothetical representative officer (%)	Difference	N
Race	White	48.64	37.65	11.00** [10.75, 11.24]	69,620
	Hispanic	28.46	29.89	−1.43** [−1.64, −1.21]	40,739
	Black	15.09	18.75	−3.66** [−3.84, −3.48]	21,599
	Other/unknown race	1.71	3.41	−1.69** [−1.76, −1.63]	2,452
	Asian	6.09	10.31	−4.23** [−4.35, −4.10]	8,711
Party (voting age population)	Republican	36.91	14.88	22.04** [21.79, 22.28]	52,831
	Democratic	30.62	41.66	−11.04** [−11.27, −10.80]	43,821
	Other/unknown party	32.47	43.47	−11.00** [−11.24, −10.76]	46,469
Turnout (voting age population)	General election, 2020	74.59	55.14	19.46** [19.23, 19.68]	105,337
Gender	Male	89.36	48.65	40.71** [40.55, 40.87]	127,895
	Female	10.64	51.35	−40.71** [−40.87, −40.55]	15,226
Age (years)	–	43.00	37.51	7.04** [6.96, 7.12]	131,864
Household income ($)	–	116738.99	94942.25	21 796.75** [21461.31, 22132.18]	130,898

Table I.5: **Officer Traits Relative to Jurisdictions (Estimated Bounds for Officers with Multiple Matches).** The table displays, from left to right, the lowest possible share of officers with a given attribute; the largest share of officers with a given attribute; the share of officers who would have the attribute if taken as a random draw from their jurisdictions; and the differences between this hypothetical share and the lower and upper bounds. Lower and upper bounds are computed by, e.g., assuming that an officer is Democratic if even one of their multiple L2 matches fits this description. **denotes $p<.01$; * denotes $p<.05$.

Variable	Value	Officer lower bound (%)	Officer upper bound (%)	Hypothetical representative officer (%)	Difference lower bound	Difference upper bound
Race	White	50.66	51.60	37.95	12.71^{**}	13.65^{**}
	Hispanic	23.66	23.84	27.98	-4.31^{**}	-4.13^{**}
	Black	15.80	16.58	21.26	-5.46^{**}	-4.68^{**}
	Other/unknown race	3.59	3.79	3.42	0.17^{**}	0.37^{**}
	Asian	5.28	5.35	9.40	-4.11^{**}	-4.04^{**}
Party (voting age population)	Republican	23.52	48.55	14.11	9.42^{**}	34.44^{**}
	Democratic	18.52	45.44	43.50	-24.98^{**}	1.94^{**}
	Other/unknown party	27.26	52.45	42.64	-15.38^{**}	9.81^{**}
Turnout (voting age population)	General election, 2020	53.38	77.98	54.62	-1.25^{**}	23.35^{**}
Age (years)	–	36.00	50.00	36.84	1.17^{**}	15.66^{**}
Household income ($)	–	90855.54	146160.98	92220.54	-1365.00^{**}	53940.44^{**}

Table I.6: **Name-only and Name/Age Matching in Officer-Civilian Trait Comparisons.** Comparisons based on full name only (top panel) and based on both full name and age (bottom panel) are shown for the 20 agencies with officer age available. The table displays, from left to right, the attributes of actual officers (means, except where otherwise noted) with a given attribute; the attributes of a hypothetical set of officers randomly drawn from their respective jurisdictions; and the difference between the two. Census data does not provide means or full distributions for age; we therefore report, median of actual officer ages, median age for a hypothetical set of officers with ages equal to the median age in their jurisdiction, and the difference in means between the two. **denotes $p<.01$; * denotes $p<.05$; brackets contain 95% confidence intervals. N indicates the number of officers.

Variable	Value	Actual Officer %	Hypothetical Representative Officer %	Difference	N
Race	White	54.71	38.69	16.02** [15.53, 16.52]	20,216
	Hispanic	19.63	24.72	-5.09** [-5.48, -4.69]	7,255
	Black	21.85	28.12	-6.27** [-6.68, -5.85]	8,073
	Other/unknown race	1.40	2.58	-1.18** [-1.30, -1.06]	517
	Asian	2.41	5.90	-3.49** [-3.65, -3.33]	890
Party (voting age population)	Republican	27.23	10.95	16.28** [15.84, 16.72]	10,062
	Democratic	41.62	49.38	-7.75** [-8.25, -7.26]	15,380
	Other/unknown party	31.15	39.67	-8.52** [-9.00, -8.04]	11,509
Turnout (voting age population)	General election, 2020	73.07	55.47	17.60** [17.13, 18.06]	27,000
Gender	Male	80.42	48.31	32.10** [31.70, 32.51]	29,715
	Female	19.58	51.69	-32.10** [-32.51, -31.70]	7,236
Age (years)	-	43.00	35.94	8.36** [8.20, 8.51]	33,337
Household income ($)	-	103639.23	80704.08	22935.15** [22319.33, 23550.96]	33,030

(a) **Using name only**

Variable	Value	Actual Officer %	Hypothetical Representative Officer %	Difference	N
Race	White	54.68	38.69	15.99** [15.50, 16.48]	20,204
	Hispanic	19.63	24.72	-5.09** [-5.48, -4.69]	7,255
	Black	21.85	28.12	-6.27** [-6.68, -5.85]	8,074
	Other/unknown race	1.43	2.58	-1.15** [-1.27, -1.03]	528
	Asian	2.41	5.90	-3.49** [-3.65, -3.33]	890
Party (voting age population)	Republican	28.56	10.95	17.61** [17.16, 18.06]	10,555
	Democratic	41.48	49.38	-7.90** [-8.39, -7.41]	15,326
	Other/unknown party	29.96	39.67	-9.71** [-10.18, -9.24]	11,070
Turnout (voting age population)	General election, 2020	74.91	55.47	19.44** [18.98, 19.89]	27,680
Gender	Male	80.42	48.31	32.10** [31.70, 32.51]	29,715
	Female	19.58	51.69	-32.10** [-32.51, -31.70]	7,236
Age (Years)	-	43.00	36.01	7.01** [6.89, 7.13]	34,095
Household income ($)	-	105576.41	80699.28	24877.12** [24273.29, 25480.95]	32,897

(b) **Using name and age**

Table I.7: **Name-only and Name/Date-of-Birth Matching in Officer-Civilian Trait Comparisons.** Comparisons based on full name only (top panel) and based on both full name and date-of-birth (bottom panel) are shown for the three agencies with officer age available. The table displays, from left to right, the attributes of actual officers (means, except where otherwise noted) with a given attribute; the attributes of a hypothetical set of officers randomly drawn from their respective jurisdictions; and the difference between the two. Census data does not provide means or full distributions for age; we therefore report, median of actual officer ages, median age for a hypothetical set of officers with ages equal to the median age in their jurisdiction, and the difference in means between the two. **denotes $p<.01$; * denotes $p<.05$; brackets contain 95% confidence intervals. N indicates the number of officers.

Variable	Value	Actual Officer %	Hypothetical Representative Officer %	Difference	N
Race	White	45.76	33.97	11.79** [11.00, 12.58]	6,693
	Hispanic	31.05	32.07	-1.02** [-1.73, -0.32]	4,541
	Black	17.62	25.85	-8.23** [-8.84, -7.62]	2,577
	Other/unknown race	2.50	2.20	0.29* [0.04, 0.55]	365
	Asian	3.08	5.90	-2.83** [-3.10, -2.55]	450
Party (voting age population)	Republican	16.70	5.81	10.89** [10.30, 11.48]	2,442
	Democratic	53.85	52.99	0.86* [0.07, 1.66]	7,876
	Other/unknown party	29.45	41.21	-11.75** [-12.49, -11.02]	4,308
Turnout (voting age population)	General election, 2020	75.65	52.43	23.22** [22.52, 23.91]	11,065
Gender	Male	78.02	48.68	29.34** [28.66, 30.01]	11,411
	Female	21.98	51.32	-29.34** [-30.01, -28.66]	3,215
Age (years)	-	44.00	35.36	9.06** [8.85, 9.28]	13,870
Household income ($)	-	104670.96	84678.77	19992.20** [19053.12, 20931.28]	13,676

(a) **Using name only**

Variable	Value	Actual Officer %	Hypothetical Representative Officer %	Difference	N
Race	White	45.58	33.97	11.61** [10.81, 12.40]	6,666
	Hispanic	30.99	32.07	-1.08** [-1.78, -0.37]	4,533
	Black	17.65	25.85	-8.20** [-8.81, -7.59]	2,582
	Other/unknown race	2.70	2.20	0.50** [0.24, 0.76]	395
	Asian	3.08	5.90	-2.83** [-3.10, -2.55]	450
Party (voting age population)	Republican	16.01	5.81	10.20** [9.62, 10.78]	2,341
	Democratic	49.56	52.99	-3.42** [-4.22, -2.63]	7,249
	Other/unknown party	34.43	41.21	-6.78** [-7.54, -6.01]	5,036
Turnout (voting age population)	General election, 2020	69.00	52.43	16.57** [15.82, 17.32]	10,092
Gender	Male	78.02	48.68	29.34** [28.66, 30.01]	11,411
	Female	21.98	51.32	-29.34** [-30.01, -28.66]	3,215
Age (years)	-	44.00	35.34	8.07** [7.89, 8.24]	11,827
Household income ($)	-	108107.45	84525.80	23581.65** [22594.68, 24568.62]	11,729

(b) **Using name and date of birth**

J Balance Tests for Behavioral Analysis in Chicago

We conduct a series of balance tests to validate that we are comparing officers working in common circumstances in the Chicago behavioral analysis. We merged our Chicago behavioral data with incident-level data on crimes reported from the city's open-data portal for beats where geographic location was available. Specifically, we paired each officer shift with the number of reported incidents of each category in the time and location of each officer shift. We then code these incidents based on whether they were likely non-discretionary (i.e., initiated by civilians, as opposed to officers) based on Table 4 of Abdul-Razzak and Hallberg (2022). The logic of this test is that imbalance in the number of discretionary incidents may be an effect of an officer's deployment (and are thus not used in this test) but imbalance in non-discretionary incidents would indicate that our research design failed to hold circumstances fixed. We estimate separate OLS models predicting the propensity of a Democratic officer to be assigned as a function of the number of non-discretionary crimes of a given category, with MDSB fixed effects. Standard errors are clustered by officers. Coefficients indicate change in the propensity score given a one-unit increase in a crime. Raw p values and BH-corrected p-values are displayed for each test. Table J.1 shows that no crime variables predict deployment of a Democrat after a multiple testing correction.

Table J.1: **Balance Tests Predicting Deployment of Democrat.** The table displays the coefficients on crime counts from individual OLS regressions with MDSB fixed effects predicting the deployment of a Democratic officer. No crimes are predictive of deployment of a Democrat after a multiple testing correction, consistent with as-if random assignment of officers within MDSBs.

Crime	Coef.	Raw p value	BH-corrected p value
Forgery counterfeiting	0.014	0.048	0.334
Vandalism	0.002	0.266	0.884
Sexual assault	−0.003	0.660	0.884
Sexual abuse	0.002	0.789	0.884
Murder	−0.002	0.884	0.884
Manslaughter	−0.063	0.569	0.884
Burglary	0.001	0.570	0.884
